# Bioassay-guided isolation and structural characterization of antimicrobial phytoconstituents from *Limonia acidissima* L. leaves and evaluation of antimicrobial, anthelmintic, and cytotoxic effects: an *in vitro* and *in silico* approach

**DOI:** 10.3389/fcimb.2026.1920478

**Published:** 2026-09-11

**Authors:** Sujogya Kumar Panda, Ajmal Khan, Masoud Besati, Mahdi Yaghoobi, Haibo Hu, Jan Paeshuyse, Liliane Schoofs, Walter Luyten

**Affiliations:** 1 Department of Biology, Animal Physiology and Neurobiology Section, KU Leuven, Leuven, Belgium; 2 Centre for Biotechnology, School of Pharmaceutical Sciences, Siksha ‘O’ Anusandhan (Deemed to be University), Kalinga Nagar, Bhubaneswar, India; 3 Department of Zoology, University of Swat Charbagh, Swat, Khyber Pakhtunkhwa, Pakistan; 4 Department of Pharmaceutical Engineering, Medicinal Plants and Drugs Research Institute, Shahid Beheshti University, Evin, Tehran, Iran; 5 National Engineering Research Center for Modernization of Traditional Chinese Medicine - Hakka Medical Resources Branch, School of Pharmacy, Gannan Medical University, Ganzhou, China; 6 Department of Biosystems, Animal and Human (A2H), Katholieke Universiteit (KU) Leuven, Leuven, Belgium

**Keywords:** bioassay guided isolation, biological evaluation, *Limonia acidissima* L., medicinal plant, molecular docking

## Abstract

**Introduction:**

Rising antimicrobial and antiparasitic resistance drives the need for novel bioactive compounds from medicinal plants, a key source of therapeutic natural products. *Limonia acidissima* is widely used in traditional medicine, but its leaves are underexplored despite the reported pharmacological properties of other plant parts. This study evaluated the antimicrobial potential of *L. acidissima* leaves using a bioassay-guided approach and assessed the antimicrobial, anthelmintic, and cytotoxic properties of the isolated compounds, supported by molecular docking and *in silico* analyses.

**Methods:**

Leaf extracts of *L. acidissima* were prepared using acetone, ethanol, and water and screened against representative microbes. The most active acetone extract was selected for bioassay-guided fractionation using silica gel chromatography and HPLC. Structural characterization was performed using UHPLC-MS/MS and NMR spectroscopy. The isolated compounds were subsequently evaluated in relevant biological assays and *in silico* assessment.

**Results and discussion:**

Bioassay-guided isolation yielded four compounds, tentatively identified as 2,2′-dihydroxy-4,7,4′,7′-tetramethoxy-1,1′-biphenanthrene (C1), kaempferol (C2), luteolin (C3), and kaempferol-3-*O*-α-L-rhamnopyranoside or afzelin (C4). All isolated compounds exhibited significant antimicrobial activity against Gram-positive bacteria, with comparatively limited activity against Gram-negative bacteria and yeast. They also showed moderate anthelmintic activity and selective cytotoxicity. Docking analysis revealed that C4 showed the strongest and most consistent binding across most targets, while C3 also exhibited high affinity, particularly against microbial receptors. C2 demonstrated moderate to strong activity, especially against *S. aureus*, whereas C1 showed generally weak binding. Overall, kaempferol-3-*O*-α-L-rhamnoside (C4) and luteolin (C3) emerged as the most promising candidates for further biological evaluation.

**Conclusion:**

This study underscores that *L. acidissima* leaves are a potential source of bioactive compounds with multifunctional biological activities. Notably, C1, C3, and C4 may be reported for the first time from this plant, and several biological activities, together with molecular docking analyses, are described for this species. These findings support the traditional medicinal use of plants and reinforce the importance of a bioassay-guided approach and *in silico* studies in the discovery of potential therapeutic agents.

## 1 Introduction

Antimicrobial resistance has emerged as a challenging public health issue shortly after the discovery of the first antibiotic. The rapid development of bacterial resistance to a range of antibiotics has prompted researchers to explore novel antimicrobial agents that could replace existing antibiotics and combat multidrug resistance (Angelini, 2024). Antiparasitic drug resistance has also been reported around the globe, with a significant burden on human health (Haraguchi et al., 2024). Humans have relied on medicinal plants to treat various diseases since prehistoric times, and these plants were their only option for recovery from disease in their environments (Jamshidi-Kia et al., 2018). Traditional medicinal plants act as a rich source of natural products that have been used for ages to treat various diseases. Natural products exhibit a rich diversity of structures and pharmacological properties, and numerous effective natural products have been isolated from plants. The knowledge associated with traditional herbal medicine has opened the way for the exploration of novel and effective pharmaceutical products (Nasim et al., 2022). Natural products of plant origin are potentially safe and affordable alternatives to antibiotics due to their broad pharmaceutical applications (Parvez and Sarker, 2021).

*Limonia acidissima* L. (wood apple or elephant apple), a member of the family Rutaceae and subfamily Citroideae or Aurantiodeae, is the only member of the genus *Limonia*, and is attributed multiple medicinal properties (Sharma and Tenguria, 2021). It is a multi-stemmed tree growing to 9 m tall. It is found in tropical and subtropical regions worldwide, including the Indian subcontinent, Sri Lanka, and Southeast Asia. It is a famous traditional medicinal plant with rough or spiny bark, pinnate leaves, and berry-shaped fruits. The leaves have 5–7 leaflets, measuring 25–35 mm long and 10–20 mm wide. When crushed, the leaves give off a citrus scent. The fruit has a diameter of 5–9 mm and has a sweet or sour taste (Banerjee et al., 2011). *L. acidissima* is a famous traditional medicinal plant in many communities around the world (Daphedar et al., 2024). Traditionally, various parts of this plant, including roots, fruits, bark, and leaves, are used for various therapeutic purposes (Pandey et al., 2014). The fruits are used to treat tumors, wounds, insect bites, as well as liver, heart, gastric, respiratory, gum, and eye-related diseases. The leaves are used to treat hiccups, skin infections, heart disease, urinary tract disorders, constipation, indigestion, vomiting, and dysentery. The roots, fruits, and resin are used in some preparations to treat diabetes in Sri Lankan Siddha Medicine (Sathasivampillai and Sebastian, 2021).

*L. acidissima* contains various bioactive chemicals with a range of bioactivities. Its extracts contain alkaloids, flavonoids, phenols, terpenoids, tannins, fats, steroids, saponins, glycosides, gum, mucilage, and essential oils. Its extracts possess antimicrobial, anti-dermatophyte, antioxidant, anti-inflammatory, and anticancer activities (Sujitha and Venkatalakshmi, 2021; Jamil et al., 2019). The fruit extracts contain alkaloids, flavonoids, tannins, and phenols, which exhibit various pharmacological properties, including antimicrobial activity (Daphedar et al., 2024). The fruit extracts have been reported to have wound-healing, antioxidant, and antimicrobial activities (Ilango and Chitra, 2010). The ethanol extracts of its leaves have been reported to contain phytoconstituents, including alkaloids, anthraquinones, terpenoids, and saponins, and to exhibit potential antibacterial activity against Gram-positive and Gram-negative bacteria (Jamil et al., 2019). The petroleum ether extracts of its leaves have also been reported to have antibacterial activity against *S. aureus* and *E. coli* (Harshali et al., 2015). The different parts of this plant species have been reported to contain multiple compounds, but the isolation, characterization, and structural elucidation of these bioactive compounds have not been extensively investigated, particularly in the leaves. Although several chemical constituents have previously been isolated from the leaves of *L. acidissima* (syn. *Feronia limonia*), including flavonoids and furanocoumarins such as orientin, vitexin, psoralen, bergapten, and xanthotoxin (Intekhab and Aslam, 2009), and various coumarins, flavonoids, alkaloids, and sterols have been reported from other plant parts (Rahman and Gray, 2002; Pitchai et al., 2012), the bioassay-guided isolation of antimicrobial constituents from *L. acidissima* leaves and their subsequent integrated evaluation for antimicrobial, anthelmintic, and cytotoxic activities, supported by molecular docking, remains limited. The present study therefore extends previous phytochemical investigations by linking the biological activity of the leaf extract to specific isolated constituents and evaluating their multifunctional biological potential through complementary *in vitro* and *in silico* approaches.

Bioassay-guided fractionation provides a systematic strategy for identifying biologically active constituents within complex plant extracts by linking successive separation and purification steps with biological activity testing (Nothias et al., 2018). This approach enables the prioritization of active fractions for subsequent purification and structural characterization, particularly when the bioactive constituents are not known in advance (Meunier et al., 2024). Thus, this study was designed to investigate the bioactivity of *L. acidissima* leaf extracts and their fractions prepared in different solvents, and to report their antimicrobial activities against common infectious agents, including representative species of Gram-positive and Gram-negative bacteria and fungal pathogens. In addition, this study aims to address a gap in the bioassay-guided approach by evaluating the *in vitro* antimicrobial, anthelmintic, and cytotoxic activities of bioactive compounds isolated from *L. acidissima*, along with *in silico* assessments.

## 2 Materials and methods

The experimental procedures include extract preparation, antimicrobial activity testing, and bioassay-guided chromatographic approaches, including silica gel column chromatography, high-performance liquid chromatography (HPLC), and mass spectrometry (MS) for compound separation and identification. The isolated compounds were evaluated for their antimicrobial, anthelmintic, and cytotoxicity potential.

### 2.1 Chemicals and reagents

The chemicals and reagents used in this study were largely the same as those described in our previous work (Khan et al., 2025b). For clarity and reproducibility, the details are provided below.

HPLC-grade acetone, n-hexane, ethyl acetate, methanol, and acetonitrile were supplied by Sigma-Aldrich (St. Louis, MO, USA). Absolute ethanol was obtained from Fischer Chemicals (Loughborough, Leicestershire, UK). Sterile deionized water used throughout the study was generated using a Milli-Q Reagent Water System (Merck Millipore, Burlington, MA, USA). Yeast extract and Bacto™ peptone were purchased from Lab M Ltd. (Lancashire, UK).

The following chemicals and reagents were acquired from Sigma-Aldrich (St. Louis, MO, USA): molecular biology grade dimethyl sulphoxide (DMSO), dextrose, sodium chloride, sodium hydroxide, magnesium sulphate, potassium hydrogen phosphate, potassium dihydrogen phosphate, disodium hydrogen phosphate, ciprofloxacin, miconazole nitrate salt, gossypol, levamisole hydrochloride, fetal bovine serum (FBS), trypsin/EDTA, Dulbecco’s Modified Eagle Medium-high glucose (DMEM), phosphate-buffered saline (PBS), RPMI-MOPS medium, and penicillin-streptomycin solution used for cell culture assays. Resazurin salt was procured from Acros Organics (Geel, Belgium).

### 2.2 Plant material

Fresh leaves of *L. acidissima* Groff were collected during a field trip in Phasi, Ganjam, India, in August 2020. The plant material was taxonomically authenticated by Prof. Dr. Pratap Chandra Panda of the Center for Biotechnology, Siksha ‘O’ Anusandhan (Deemed to be University), Bhubaneswar, India. A voucher specimen (2248/CBT) was deposited in the herbarium of the same institution. The leaves were dried in the absence of sunlight for 7 days at ambient temperature. The leaves were ground into a powder using an electric grinder, then packed in plastic bags and transported to KU Leuven, Belgium, in accordance with the standard export and import protocols of both countries.

### 2.3 Plant extract preparation

Bioactive compounds were extracted from *L. acidissima* leaves using both small- and large-scale methods. Small-scale extraction was conducted for initial screening and selection of the best extractant, whereas large-scale extraction was conducted to obtain sufficient crude extract for bioassay-guided fractionation analyses. The extraction methodologies are described below.

#### 2.3.1 Small-scale extraction

Small-scale extraction was carried out using acetone, ethanol, and water to compare extraction efficiency and perform preliminary bioactivity screening. The method was generally consistent with our previous work (Khan et al., 2025b), with slight adjustments as needed. In brief, 1 g of powdered plant material was weighed and placed into three separate sterile 15 mL Falcon tubes, each containing 10 mL of the respective solvent. The samples were well mixed and then subjected to repeated vortexing and sonication using a Branson Ultrasonics water bath sonicator (Danbury, CT, USA) for 15 minutes per cycle, repeated four times with 6-hour intervals to improve extraction. After sonication, the tubes were centrifuged at 3500 rpm for 10 minutes. The supernatants (1 mL) were carefully transferred into 1.5 mL Eppendorf tubes and dried using a Savant SpeedVac concentrator (FTS Systems Inc., Stone Ridge, NY, USA) until complete solvent removal. The dried extracts were then weighed and re-dissolved to a final concentration of 20 mg/mL, using DMSO for acetone and ethanol extracts, and sterile water for aqueous extracts. All extracts were stored at 4 °C until further analysis, in accordance with previously reported procedures (Khan et al., 2025b; Panda et al., 2017).

#### 2.3.2 Large-scale extraction

**Figure 1:**
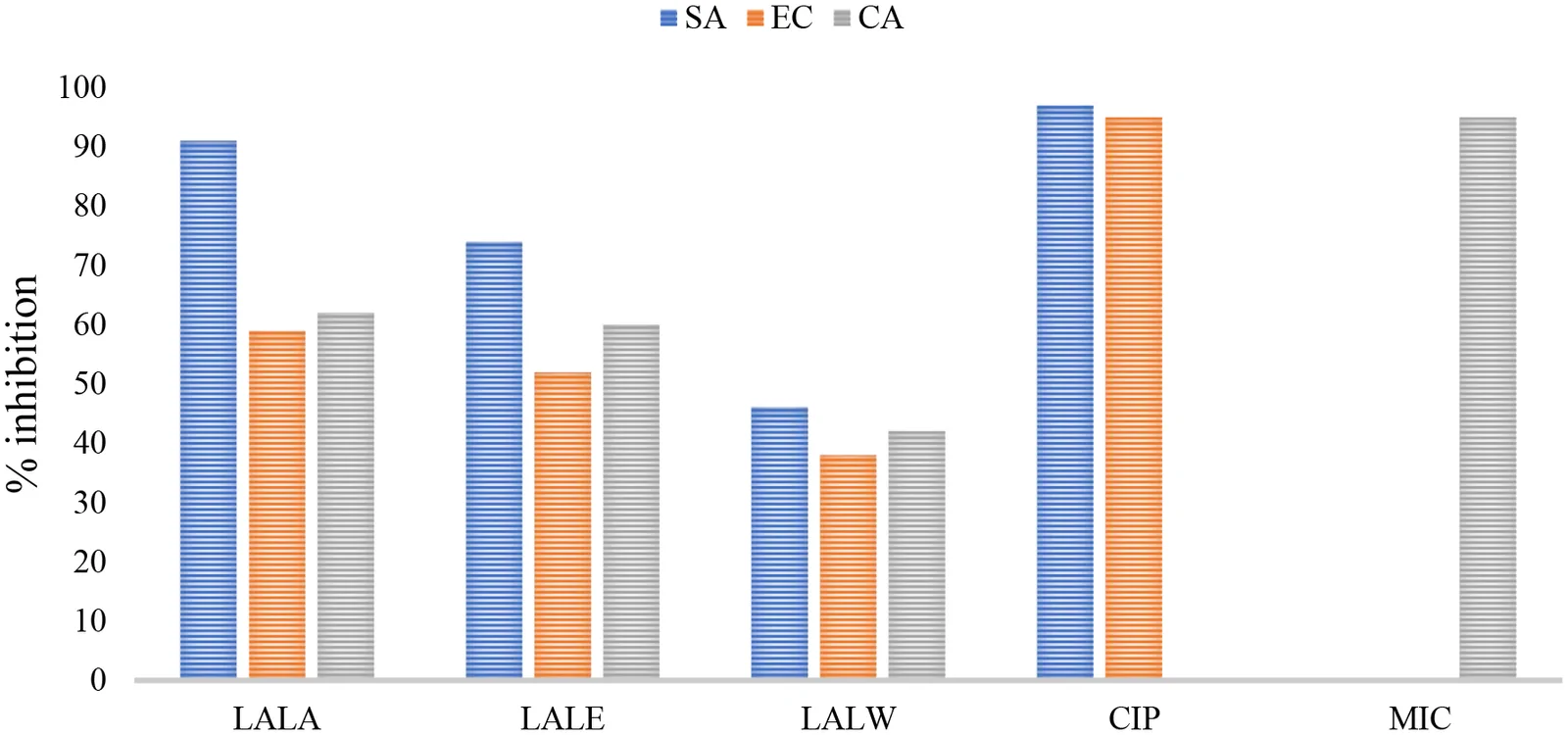
Antimicrobial activity of the crude extracts of *L. acidissima* leaves (LAL) (20 mg/mL stock; final concentrations: 1000 µg/mL for bacteria and 400 µg/mL for yeast). SA, *Staphylococcus aureus*; EC, *Escherichia coli*; CA, *Candida albicans*; LA, *Limonia acidissima*; L, leaves; A, acetone; E, ethanol; W, water; CIP, ciprofloxacin (positive control for *S. aureus* and *E. coli*)); MIC, miconazole (positive control for *C. albicans*).

The large-scale extraction was carried out in acetone based on the pronounced bioactivity of this extract observed during preliminary screening. As shown in **Figure 1**, the acetone extract exhibited the highest antimicrobial activity against all three tested microorganisms (*S. aureus*, *E. coli*, and *C. albicans*) compared with the ethanol and aqueous extracts. Therefore, acetone was selected for large-scale extraction and subsequent bioassay-guided fractionation. For large-scale extraction, 50 grams of powdered *L. acidissima* plant material were added to 0.5 L of acetone in a screw-capped glass bottle, then thoroughly shaken and sonicated four times for 30 minutes at 6-hour intervals, as described earlier in the small-scale extraction section. The extract supernatant was then filtered through a Whatman Grade 1 filter paper (18.5 cm diameter), and the solvent was removed under reduced pressure using a Rotavapor (B-100, Buchi^®^, Flawil, Switzerland) in a pre-weighed round-bottom flask. The process was repeated twice, and finally, the sediments were compacted by centrifugation at ambient temperature for 20 minutes. The combined dried extracts were then redissolved in the original solvent in a round-bottom flask, and a thick slurry was prepared by adding about 10 g of silica gel (63-200 µm) for every 10 g of dried extracts. The slurry mixture was then dried by evaporation for dry-loading onto a silica gel column. The mixture was then transferred from a conical flask to a 50 mL plastic tube with a screw cap (Liu et al., 2018) for storage until use.

### 2.4 Bioassay-guided chromatographic and spectroscopic analysis for isolation of compounds from *L. acidissima*

A bioassay-guided approach was used to select the most active constituents of *L. acidissima* extracts by continuously correlating bioactivity with successive fractionation steps. The purification of active components was achieved by silica gel column chromatography followed by HPLC.

#### 2.4.1 Fractionation of extracts by silica gel column chromatography

The large-scale purification and fractionation of the selected active acetone extract from the leaves of *L. acidissima* were performed using silica gel column chromatography to separate fractions based on polarity and adsorption, following the protocol of Hu et al. (2021). A silica gel column (ECOPLUS 35×500 mm, YMC, Kyoto, Japan) was connected to a preparative liquid chromatography set-up (Waters Delta 600 multi-solvent quaternary pump, Waters detector 2487, Waters 600 controller, Massachusetts, USA), with a mobile phase consisting of hexane (A), ethyl acetate (EtOAc) (B), methanol (MeOH) (C) and 20% acetic acid in methanol (D). The flow rate was maintained at 40 mL/min with a step gradient from 95% A and 5% B to 100% D (5-20% step every 10 min). In short, a glass column (30 cm by 8 cm) was packed with 200 g of silica gel (63-200 µm), suspended in 500 mL of 100% hexane to form a slurry. After the silica gel had settled, the dried extract adsorbed to the silica gel was evenly distributed on the surface of the silica bed, and sea sand was added to cover the sample. The step gradient started from hexane:EtOAc (95:5, 90:10, 80:20, 70:30, 60:40, 50:50, 40:60, 30:70, 20:80, 10:90, 0:100), followed by EtOAc: MeOH (80:20, 60:40, 40:60, 20:80, 0:100) and finally MeOH:20% acetic acid in MeOH (75:25, 50:50, 25:75, 0:100). The eluate was monitored using an absorbance detector (Dual λ, Waters detector 2487) at wavelengths of 360 nm and 254 nm. Subsequently, 200 fractions were collected at 1-minute intervals in 50 mL Falcon tubes with screw caps. One mL from each collected fraction was transferred to Eppendorf tubes and dried in a Savant SpeedVac Concentrator (FTS Systems Inc., Stone Ridge, NY, USA). The dried fractions were then dissolved in 100 μL DMSO for bioactivity testing. Based on the observed bioactivities, the active fractions were grouped, and the most active fractions within each group were selected and subjected to HPLC for further separation (Hu et al., 2021). Fractions were considered active based on their antimicrobial activity against the tested microorganisms, with particular attention given to those showing broad-spectrum activity. Among the active fractions, those showing the highest percentage inhibition were prioritized for further purification. Where applicable, IC_50_ values were also considered, with fractions exhibiting lower IC_50_ values prioritized as more potent candidates for subsequent chromatographic separation and compound isolation.

#### 2.4.2 HPLC-based sub-fractionation of active fractions

The selected active fractions from silica gel column chromatography were then analyzed by HPLC (high-performance liquid chromatography) using an LC-20AT pump, an SPD-M20A detector, and a DGU-20A3R degasser (SHIMADZU, Kyoto, Japan) with a diode-array detector (DAD). The HPLC chromatograms were analyzed using LabSolutions software. Before the run, 1 mL of the dried active fraction was dissolved in a mixture of acetonitrile (ACN) and water using the lowest possible ACN concentration required to achieve complete dissolution. The solution was sonicated and centrifuged for 30 seconds, then filtered through an H-PTFE filter (0.45 µm pore size). Afterward, 100 µL of the sample was injected into the HPLC system for separation on a semi-preparative C18 column (250×10mm, 5 μm, C18, Sunfire, Waters), ensuring that the amount did not exceed the equivalent of 2 mg of sample to avoid column overloading. MilliQ water (A) and ACN (B) (each with 0.1% TFA) were used for gradient elution. After optimizing the HPLC conditions, a gradient was used as listed in **Supplementary Table 1**, starting at 0% sample and 100% ACN, with a flow rate of 4 mL/min. Every minute, the eluate fractions were collected and later dried in a SpeedVac Concentrator. Afterward, 12 µL DMSO was added to dissolve the samples, and the bioactivity tests were performed. An activity with more than 50% inhibition was considered significant. When needed, the separation of corresponding active peaks was optimized using analytical columns (250×4.6mm, 5 μm, C18, Symmetry, Synergi, Phenomenex; 250×4.6mm, 4 μm, Hydro-RP, Synergi, Phenomenex; Waters; 150×4.6mm, 4 μm, Polar-RP). The final active peaks were then collected, dried, weighed, and their antimicrobial, anthelmintic, and cytotoxicity activities were evaluated. After verification of bioactivities, the peaks were collected and stored at 4 °C for UHPLC-MS/MS analysis (Hu et al., 2023).

### 2.5 UHPLC-MS/MS and NMR spectroscopy-based identification of active compounds

The identification of active compounds was performed using UHPLC-MS/MS and NMR spectroscopy. The UHPLC-MS/MS analysis was carried out with an Ultimate 3000 UHPLC system (Dionex Thermo Scientific) equipped with a C18 column (2 µm, 100 Å, 50 µm × 15 cm, Easy-Spray, Thermo Scientific, USA) and an ultra-high resolution Orbitrap Elite hybrid MS spectrometer (Thermo Scientific, USA). The UHPLC was set to a flow rate of 0.3 µL/min, a column temperature of 35 °C, and an injection volume of 15 µL. The mobile phases consisted of water (eluent A) and 100% acetonitrile (eluent B), both containing 0.1% TFA. The step gradients were: 0–5 min, 5% B; 5–20 min, 5% to 100% B; 20–23 min, 100% B; 23–24min, 100% to 5% B; and 24–34 min, 5% B to re-equilibrate the system. For MS detection, the Orbitrap-Ion Trap MS spectrometer was fitted with a heated electrospray ionization (ESI) ion source, and both positive and negative modes were acquired in full-scan mode over m/z 100–2000. For structural identification of compounds, the top 20 ions’ MS/MS fragmentation (dd-MS^2^-TOP 20) was performed over the m/z range of 50–2000. The MS parameters were optimized based on the conditions adopted from Hu et al. (2021): spray voltage: -2.1 kV to +2.1 kV, capillary temperature: 275 °C, multipole RF amplifier: 800 Vp-p, s-lens RF level: 60%, and reagent ion source temperature: 160 °C. The stepped ion trap mass spectrometry (ITMS) was set to 35eV to obtain MS/MS spectra of the most intense ions from Fourier transform mass spectrometry (FTMS).

The mass spectrometry data were analyzed using the ACD/MS Workbook Suite 2023 software, in combination with MS Fragmenter and Chromgenius (ACD/Labs, Canada). The tentative identification of compounds was based on accurate mass measurements, MS^1^ molecular ions, and MS^2^ fragmentation patterns. Firstly, each UHPLC-MS data set was imported into Xcalibur and subtracted for the blank solvent in both positive and negative ion modes to exclude contaminants from the solvent, tube, column, or background. Secondly, the MS^2^ data for each compound were compared with fragmentation patterns to identify structures using databases such as the NIST mass spectral library, mzVault, and mzCloud. Thirdly, the MS data were imported into Workbook Suite, and the ITA (IntelliTarget Analysis) and IX 2.0 (IntelliXtract) functions were applied to the targeted and untargeted algorithms, with an accuracy limit of 5 ppm for both. In addition to commercial spectral databases, the COCONUT database and a manually developed *L. acidissima* database (sourced from PubChem, relevant literature, and other databases), both linked to ACD/Labs software, were used to confirm compound identification. Finally, MS Fragmenter was used to predict fragmentation patterns, and Chromgenius was used to calculate retention time (tR) via chemical similarity searches. Based on these steps, the spectra of all the compounds were interpreted and verified, and the compounds were identified.

Proton nuclear magnetic resonance (^1^H NMR) spectroscopy was used to help identify the compounds reported for the first time from this plant species, following the methods described by Liu et al. (2018). ^1^H NMR analysis provides abundant information about the chemical environments of hydrogen atoms within a molecule, and is a powerful tool for confirming molecular structure, functional groups, and purity. The samples were dissolved in deuterated MeOH, and the NMR spectra were recorded on a 400 MHz CryoFITNMR spectrometer (Bruker Advance III HD 400, USA). Chemical shifts (δ) are expressed in parts per million (ppm) using tetramethylsilane (TMS) as an internal standard. The coupling constants J are expressed in Hz, while the signal multiplicities are expressed as s (singlet), d (doublet), t (triplet), q (quartet), m (multiplet), or br (broad). The spectra were analyzed using MestReNova (Mnova) software, and the interpretation was based on chemical shifts, signal multiplicities, coupling constants, and integration values. The structure of the compounds was compared with literature reports for confirmation.

### 2.6 *In vitro* biological evaluation of extracts and compounds

The biological evaluation of the crude extracts and isolated compounds from *L. acidissima* was conducted using a series of *in vitro* assays. The evaluation included antimicrobial (antibacterial and antifungal), anthelmintic, and cytotoxic activities, all conducted using standard experimental protocols. These assays were used to systematically assess the pharmacological potential of the test samples.

#### 2.6.1 Antimicrobial assays

The antimicrobial activity of the crude extracts and compounds was assessed to determine their effectiveness against the selected microorganisms. The study included both antibacterial and antifungal assays against pathogenic and opportunistic bacterial and fungal strains of human relevance: *Staphylococcus aureus* (ATCC 65385), *Micrococcus luteus* (DPMB 3), *Escherichia coli* (ATCC 47076), *Pseudomonas aeruginosa* (PA 01), *Candida albicans* (SC 5314), and *Saccharomyces cerevisiae* (ATCC 7754). All microbial strains were obtained from the strain collection maintained and preserved at -80 °C in our laboratory at the Department of Biology, KU Leuven, Belgium. The antibacterial and antifungal assays were performed using standard protocols with minor modifications specific to the test microorganisms, as described in our previous study (Khan et al., 2025b). The results were expressed as percentage growth inhibition, as detailed in the sections below.

##### 2.6.1.1 Antibacterial assays

The antibacterial activity of the test sample was evaluated using a broth microdilution assay as described in our previous studies (Khan et al., 2025a; Khan et al., 2025b). In short, a 10 µL test sample was added to the wells of a clear, flat-bottom 96-well microtiter plate (polystyrene, Thermo Fisher Scientific), along with positive and solvent controls. Each well was then inoculated with 190 µL of a standardized microbial inoculum with an optical density (OD) of 0.003 at 620 nm. For control wells, 10 µL of extract and 190 µL of sterile LB (Luria-Bertani) broth were added to allow correction for absorption by extract components. For the positive control, 200 µg/mL (stock) of ciprofloxacin was used, and for the solvent control, 5% of DMSO or water was used. The plates were then incubated at 37 °C in a shaker-incubator for 24 hours, and were subsequently read on a Multimode Microplate Reader at 620 nm (lamp energy: 13,000) using the MikroWin 2000 software package. The OD values for wells containing a plant extract were corrected for the absorption contributed by the extract. The relative inhibition percentage (%) was calculated by dividing the difference between the OD of the test sample (A) and that of the non-inoculated extract control (B) by the average OD of the solvent control (C), then multiplying by 100.


Relative inhibition(%)=((A−B)÷C)×100

##### 2.6.1.2 Antifungal assays

The antifungal activity was assessed following the standard method described in our previous studies (Khan et al., 2025a; Khan et al., 2025b). Unlike antibacterial assays, antifungal assays used YPD instead of LB broth. In short, 4 µL of the test sample was added to each well of a clear, flat-bottom 96-well microtiter plate (polystyrene, Thermo Fisher Scientific). Then 196 µL of the diluted yeast suspension was added to the corresponding wells. Control wells were prepared with 4 µL of test sample and 196 µL of YPD broth to correct for absorption by extract components. Miconazole (250 µg/mL, stock) was used as the positive control, and 2% DMSO or water as the solvent control. The relative inhibition percentage (%) was calculated using the same formula as for the antibacterial assays.

#### 2.6.2 Anthelmintic assays

The anthelmintic activity of the test sample was assessed to determine its effectiveness against *Caenorhabditis elegans* (*C. elegans*) by following the protocol described in our previous studies (Yaghoobi et al., 2025; Liu et al., 2018; Panda et al., 2017). In short, N2 wild-type *C. elegans* were cultured at 20 °C on NGM (Nematode Growth Medium) plates with *E. Coli* OP50 and synchronized using a modified alkaline bleaching protocol. The L1 larvae were hatched overnight in S-basal buffer and then raised to the L4 stage over 48 hours. About 45 L4 larvae were seeded per well in a clear, flat-bottom 96-well microtiter plate (polystyrene, Thermo Fisher Scientific) with an *E. coli* culture and test samples, in accordance with the procedure described previously (Liu et al., 2018). The WMicroTracker system (Argentina) was used to monitor movement inhibition over a 20-hour period, using DMSO and water as solvent controls, and levamisole as a positive control.

#### 2.6.3 Cytotoxicity assays

Cytotoxicity was assessed using a resazurin-based cell viability assay with A549 (human lung epithelial tumor cells) and WI-26 VA4 (non-tumoral human lung fibroblast cells) cell lines, as per the protocol described in our previous study (Khan et al., 2025b). In short, the cell lines were maintained in a humidified 5% CO_2_ incubator in DMEM supplemented with antibiotics 100 µg/mL penicillin, 100 µg/mL streptomycin, and 10% FBS. For the test, 200 µL cell suspension (2 × 10^4^ cells per well) was plated in a clear, flat-bottom 96-well microtiter plate (polystyrene, Thermo Fisher Scientific) and incubated at 37 °C. After 24 hours, the medium was replaced, and cells in new DMEM were exposed to the test samples, DMSO or water (solvent control), and gossypol (positive/cytotoxic control; 10 mM). The plates were again incubated overnight. After 48 hours, 10 µL of resazurin solution (0.15 mg/mL in PBS, stock) was added to each well to measure cell viability. The plates were then incubated for a further 4 hours at 37 °C in a 5% CO_2_ incubator, covered with aluminum foil. The fluorescence was measured using a 550 nm excitation filter and a 590 nm emission filter in an automated multi-well fluorescence reader (FlexStation II, Molecular Devices, USA). The results were expressed as cell viability inhibition (%), calculated from fluorescence values as follows.


Cell viability inhibition(%)=100%−(treated cells−background cells)÷(DMSO controls−background controls)×100%

#### 2.6.4 Determination of IC_50_ values

The half-maximal inhibitory concentration (IC_50_) values for all bioactivity assays were determined using a two-fold serial dilution assay as described in our previous study (Khan et al., 2025a). The test samples were dissolved in DMSO or water, and a twofold serial dilution series (up to 64-fold) was prepared in a V-shaped 96-well plate (polystyrene, Thermo Fisher Scientific), followed by the corresponding biological evaluations. Percentage inhibition values obtained at each tested concentration were used to generate concentration–response curves. The X values (concentrations) were transformed using X = log(X), followed by nonlinear regression (curve fit) using the dose–response inhibition model, log(inhibitor) versus normalized response–variable slope, in GraphPad Prism version 11.0.0 (GraphPad Software, San Diego, CA, USA). The IC_50_ was defined as the concentration required to produce 50% inhibition of the measured biological response.

### 2.7 Evaluation of the *in silico* ADME

The pharmacokinetic properties of the phytoconstituents isolated from *L. acidissima* leaves were evaluated using the QikProp module integrated within the Schrödinger Maestro suite (Schrödinger, 2023). The drug-likeness of the compounds was assessed according to Lipinski’s Rule of Five, a widely accepted guideline for oral bioavailability. The screening criteria included a molecular weight of not more than 500 Da, no more than five hydrogen bond donors, no more than ten hydrogen bond acceptors, a LogP value of ≤ 5, and a molar refractivity ranging from 40 to 130. This approach was used to identify compounds with favorable ADME (Absorption, Distribution, Metabolism, and Excretion) profiles for further evaluation (Lipinski, 2004).

### 2.8 Molecular docking

Molecular docking was carried out for the compounds that successfully cleared both initial *in silico* screening filters. The selected targets represent validated or widely used proteins associated with the biological assays performed in this study, including antibacterial (*S. aureus* PBP-1b, *E. coli* PBP), antifungal (*C. albicans* CYP51), anthelmintic (*C. elegans* β-tubulin/GDP-microtubule), and cytotoxic (COX-2) activities, and were selected based on their established biological relevance and widespread use in previous molecular docking studies. In addition, the *P. falciparum* purine nucleoside phosphorylase (3PHC) receptor was included as an exploratory target to evaluate the broader interaction profile of the isolated compounds. For this investigation, the X-ray crystal structure of the enzyme was obtained from the RCSB Protein Data Bank (https://www.rcsb.org/structure/3PHC). Virtual docking of compounds was done at the binding sites of 14-α Demethylase (CYP51) from *C. albicans* (PDB ID: 5TZ1), penicillin-binding protein (PBP-1b) from *S. aureus* (PDB ID: 2Y2H), *E. coli* (PDB ID: 6F86), COX-2 enzyme (PDB ID: 1CX2), *Plasmodium falciparum* purine nucleoside phosphorylase (PDB ID: 3PHC), and *C. elegans* GDP-microtubule (PDB ID: 6E88) (Saleem et al., 2026; Ahsan et al., 2025; Cédric et al., 2024; Christalin et al., 2024; Işık et al., 2024; Assefa et al., 2023; Ali et al., 2022; Oliveira et al., 2022; Bhole et al., 2021; Bhat et al., 2015; Kumar et al., 2014; Fogel et al., 2008), using the Glide module of Schrödinger Maestro version 12.5 (Schrödinger, LLC, New York, NY, USA). The protein structures were prepared using the Protein Preparation Wizard by removing water molecules, resolving atomic conflicts, adding hydrogen atoms, and optimizing the protonation and tautomeric states of ionizable residues (Friesner et al., 2004).

The receptor grid was generated using the Receptor Grid Generation protocol in Maestro, with grid boxes of 15 × 15 × 15 or 20 × 20 × 20 Å encompassing the active site of each target. The docking calculations were performed using the Glide module, and the resulting ligand poses were ranked according to the software’s docking score (Saleem et al., 2026; Ahsan et al., 2025; Cédric et al., 2024; Christalin et al., 2024; Işık et al., 2024; Assefa et al., 2023; Ali et al., 2022; Oliveira et al., 2022; Bhole et al., 2021; Friesner et al., 2004). The docking protocol was validated based on an RMSD value of<2 Å (**Supplementary Table 2**).

### 2.9 Data processing and analysis

All biological assays and experimental measurements were performed in two independent replicate experiments (n = 2) to confirm the reproducibility of the results, following the standardized screening workflow used in this study. The reported values represent the mean of the duplicate determinations. The bioassay data were presented as percentages of inhibition, and the graphs and tables were prepared in Microsoft Excel for Microsoft 365 (Microsoft Corporation, Redmond, WA, USA). The IC_50_ values were calculated using GraphPad Prism 11.0.0 (GraphPad Software, San Diego, CA, USA). The MS data were analyzed using the ACD/MS Workbook Suite 2023 software, in combination with MS Fragmenter and Chromgenius (ACD/Labs, Canada). Molecular docking and *in silico* ADME analyses were performed using Schrödinger Maestro version 12.5 (Schrödinger, LLC, New York, NY, USA).

## 3 Results and discussion

### 3.1 Antimicrobial activity of *L. acidissima* leaf extracts

The antimicrobial activity of *L. acidissima* leaf extracts prepared in three different solvents (acetone, ethanol, and water) was investigated against *S. aureus*, *E. coli*, and *C. albicans*. As shown in **Figure 1**, the antimicrobial efficacy varies with the solvent used: acetone extracts exhibited the highest inhibitory activity against all three tested microorganisms, followed by ethanol and water extracts. The acetone extracts showed the highest antimicrobial activity with inhibition values of 91% (*S. aureus*), 59% (*E. coli*), and 62% (*C. albicans*). The inhibitory activity of the ethanol extracts was reported to be 74%, 52%, and 60% against *S. aureus*, *E. coli*, and *C. albicans*, respectively. Similarly, the water extracts showed inhibitory activity of 46% against *S. aureus*, 38% against *E. coli*, and 42% against *C. albicans*. The overall percent inhibition varied among the tested microorganisms, with *E. coli* exhibiting greater susceptibility than *S. aureus*, while the yeast *C. albicans* showed comparatively less susceptibility.

The highest antimicrobial efficacy of acetone extracts may be due to the solvent’s moderate polarity, which enables the extraction of a wide range of moderately polar compounds. The choice of solvent greatly influences the extraction of bioactive secondary metabolites; thus, the intermediate polarity of acetone enables it to extract both non-polar bioactive compounds, such as alkaloids, terpenoids, and flavonoids, and moderately polar compounds, resulting in stronger antimicrobial effects (Lee et al., 2024). Several compounds, including many antimicrobial agents, are not readily soluble in water or ethanol but are soluble in acetone; thus, their extraction varies with the solvent (Dogbey et al., 2020). Several studies have reported that acetone extracts exhibit greater antimicrobial efficacy than other solvents (Padalia and Chanda, 2015). The higher susceptibility of the tested microorganisms to the acetone extracts suggests that a wide range of phytochemicals is present. Thus, the acetone leaf extract was selected for large-scale extraction and chromatographic analysis to isolate and identify the compounds responsible for the antimicrobial activity. This selection was based on its consistently higher inhibitory activity against all three tested microorganisms compared with the ethanol and aqueous extracts, supporting its use as the starting material for subsequent bioassay-guided fractionation.

### 3.2 Bioassay-guided isolation of compounds from *L. acidissima* and their antimicrobial potential

#### 3.2.1 Antimicrobial screening of fractions obtained from silica gel column chromatography

The preliminary small-scale extraction and screening were followed by a large-scale acetone extraction of *L. acidissima* leaf material to obtain sufficient crude extract for chromatographic separation and subsequent bioassay-guided fractionation. The large-scale acetone extract was subjected to silica gel column chromatography, yielding 200 individual fractions. The chromatographic absorbance profile recorded at 360 nm and 254 nm is presented in **Supplementary Figure 1**.

**Figure 2:**
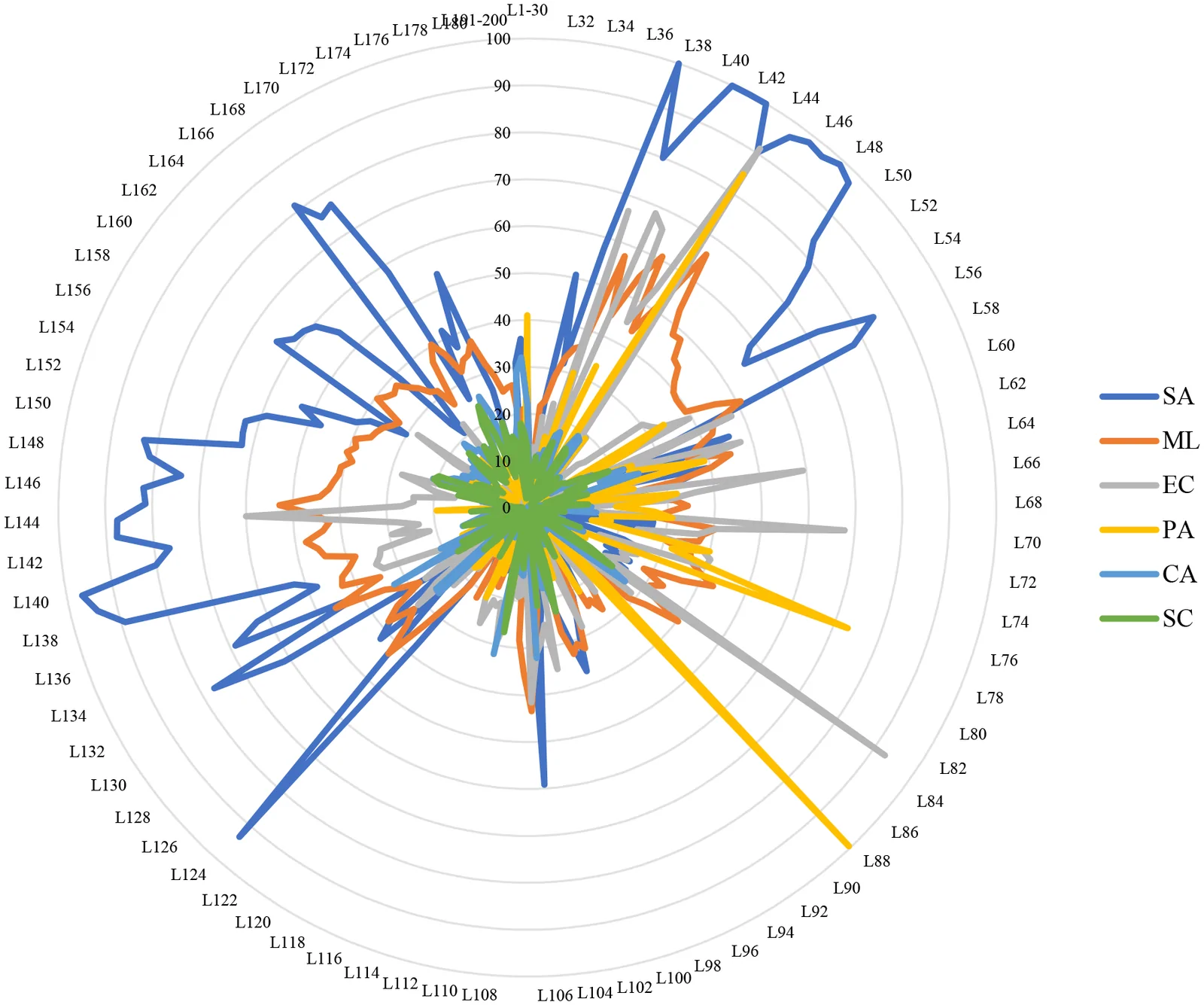
Antimicrobial activity of silica gel fractions of *L. acidissima* leaves. The scale from the center toward the outer axis (0-100) represents percentage inhibition (%). SA, Staphylococcus aureus; ML, Micrococcus luteus; EC, Escherichia coli; PA, Pseudomonas aeruginosa; CA, Candida albicans; SC, Saccharomyces cerevisiae; L, Limonia acidissima fraction.

All fractions were initially screened for antimicrobial activity against two Gram-positive bacteria (*S. aureus* and *M. luteus*), two Gram-negative bacteria (*E. coli* and *P. aeruginosa*), and two yeasts (*C. albicans* and *S. cerevisiae*). Antimicrobial activity is reported as percent inhibition values. As shown in **Figure 2**, several fractions exhibited strong inhibitory activity against the tested microorganisms, while others had little or no effect on their growth. Most of the fractions showed pronounced inhibitory activity against *S. cerevisiae*, followed by *S. aureus, E. coli, M. Luteus, P. aeruginosa*, and *C. albicans.* Moreover, the activities were observed in successive fractions, indicating that the antimicrobial activity is not limited to individual fractions but is distributed across multiple fractions that elute at different stages of the chromatographic process, depending on the solvent proportions used to extract bioactive compounds. Similarly, comparable antimicrobial activities were observed in adjacent fractions, suggesting that the same type of molecule elutes in these fractions and therefore exhibits similar activity. Thus, the adjacent active fractions were grouped into six sets of contiguous fractions (F41-F43, F56-F58, F104-F106, F123-F125, F131-F133, and F168-F170) and a single active fraction was selected from each set for further investigation.

#### 3.2.2 Antimicrobial testing of adjacent active fractions

**Figure 3:**
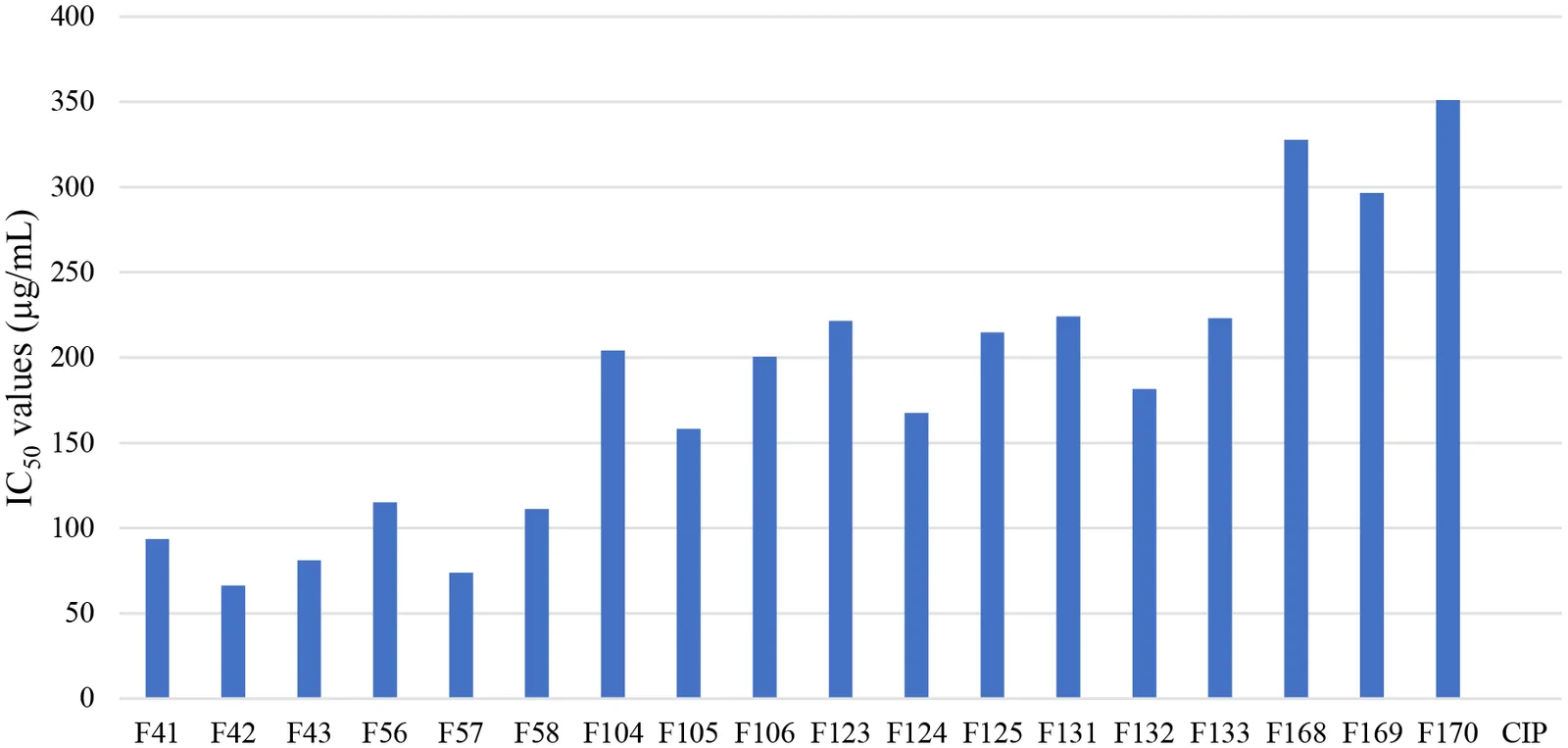
IC_50_ values of the antimicrobial activity of sets of consecutive fractions of *L. acidissima* against *S. aureus.* Ciprofloxacin (CIP), used as a positive control, showed an IC_50_ value of 0.28 µg/mL.

Based on the pronounced activity of silica gel column chromatographic fractions, the most active fraction and its adjacent fractions, i.e., F41-F43, F56-F58, F104-F106, F123-F125, F131-F133, and F168-F170, were subjected to serial dilution assays and were evaluated for their antibacterial efficacy against the representative bacterium *S. aureus*. The inhibitory effects of each fraction cluster as concentrations decrease clearly indicate concentration-dependent potency of the tested fractions (**Supplementary Figure 2**). Based on the reported growth inhibition percentages and IC_50_ values of the adjacent fractions (**Figure 3**), the most active fractions (F42, F57, F105, F124, F132, and F169) from each set were selected for further HPLC analysis.

#### 3.2.3 Evaluation of antimicrobial activity of HPLC-based sub-fractions of active fractions

Based on the results of serial dilution assays, the six most active fractions, i.e., F42, F57, F105, F124, F132, and F169, were subjected to HPLC separation by using a semi-preparative C18 column (250 × 10 mm, 5 μm, C18) for determining the specific chromatographic components or peaks responsible for the observed antibacterial activity. Among the six initially selected fractions, four exhibited pronounced antibacterial activity within individual HPLC peaks. In comparison, the other two fractions showed no pronounced activity at the individual peak level, suggesting that the observed activity in these fractions was due to synergistic effects. Thus, the four finally selected fractions with well-defined antibacterial activity, i.e., F42, F105, F124, and F169, were further evaluated for purification and tentative identification of the respective peaks using UHPLC-MS/MS. The analysis and antibacterial efficacy of the HPLC subfractions of the four selected active fractions are presented here.

##### 3.2.3.1 Fraction 42 (F42)

F42 was subjected to HPLC using a semi-preparative C18 column through a step gradient with a mobile phase of water (A) and acetonitrile (B), both containing 0.1% TFA (Trifluoroacetic acid). The HPLC run yielded 60 subfractions over 60 minutes, as evident from the chromatogram in **Supplementary Figure 3A**. The 60 subfractions were collected and tested for their antibacterial activity against *S. aureus*. The percent inhibition values of each subfraction are shown in **Supplementary Figure 3B**. The subfraction eluting at min 54 was the most active against *S. aureus*; the corresponding peak was collected and retested to confirm its antibacterial efficacy. This peak was again purified using an Analytical C18 column (250 × 4.6 mm, 5 μm, C18) and was assigned the code ‘‘C1.’’ The finally confirmed and purified active peak (C1) was then analyzed by UHPLC-MS/MS for tentative identification.

##### 3.2.3.2 Fraction 105 (F105)

F105 was also subjected to HPLC using a semipreparative C18 column with a mobile phase of water (A) and acetonitrile (B), each containing 0.1% TFA, which yielded 60 subfractions as shown in the chromatogram in **Supplementary Figure 4A**. Similarly, the antibacterial activity of the subfractions was evaluated as shown in **Supplementary Figure 4B**. The peak eluting at min 44 was purified using an Analytical C18 column and assigned the code C2. The peak C2 was further analyzed through UHPLC-MS/MS for its tentative identification.

##### 3.2.3.3 Fraction 124 (F124)

F124 was also subjected to HPLC, yielding 60 subfractions (**Supplementary Figure 5A**). Based on the antibacterial effects (**Supplementary Figure 5B**), the peak eluting at minute 31 (C3) was collected, purified, and further analyzed by UHPLC-MS/MS for tentative identification.

##### 3.2.3.4 Fraction 169 (F169)

F169 also yielded 60 subtractions upon HPLC analysis, as shown in **Supplementary Figure 6A**. Based on the percent inhibition values of the subfractions (**Supplementary Figure 6B**), the peak eluting at minute 34 (C4) was purified and processed for further analysis and chemical identification through UHPLC-MS/MS and NMR spectroscopy.

### 3.3 UHPLC-MS/MS and NMR-based structural identification of active compounds from *L. acidissima*

Based on the screening and purification of HPLC subfractions, the active peaks (C1, C2, C3, and C4) of the corresponding selected fractions of *L. acidissima* were further analyzed for obtaining the molecular mass information to support the characterization of the antimicrobial constituents using UHPLC-MS/MS in both positive and negative ion mode. In addition, NMR spectroscopy was performed for compound C4, which is reported here for the first time from *L. acidissima*. The UHPLC-MS/MS profiles of the relevant peaks showed distinct chromatographic signals with confirmed m/z (mass-to-charge) values, indicating the presence of distinct chemical constituents in each active peak. The isolated compounds corresponding to the respective peaks were tentatively identified from the mass spectrometry (MS^1^ and MS^2^) data using ACD/Labs (Advanced Chemistry Development) Software. The MS^1^ spectra were used to measure precursor ion m/z values, determine isotope distributions, and identify adducts, charge states, and neutral losses. Based on MS^1^ information, the exact mass and chemical formula of the precursor ions were generated through an auto-calculation in the ACD MS Workbook Suite coupled with its internal database, a manually built database of the *L. acidissima* plant, and the COCONUT database. The MS^2^ spectra were used for fragmentation-based structure elucidation of the candidate compounds. The MS Fragmenter was used to predict the fragmentation patterns and distinguish isomers with similar structures. The retention time calculations for different isomers were performed using ChromeGenius under the same conditions. Hence, based on MS^1^ and MS^2^ spectral information using ACD/Labs Software, the compounds were tentatively identified, and the corresponding chemical names were assigned as shown in **Table 1**.

**Table 1:** Mass and structural information of tentatively identified antimicrobial compounds from *L. acidissima*.

No.	Name	tR (min)	Formula	Ion mode	m/z calculated	m/z experimental	Difference (Da)	RDBE	MS/MS fragments
1	2,2′-dihydroxy-4,7,4′,7′-tetramethoxy-1,1′-biphenanthrene	17.976	C_32_H_26_O_6_	[M+H]^+^	507.180	507.181	0.001	19.5	332.8078 393.1889 395.2011 396.2074 506.2429 507.1809 508.1822 511.2959 579.0871
2	Kaempferol	12.848	C_15_H_10_O_6_	[M+H]^+^	287.055	287.054	-0.001	10.5	153.0253 258.0512 259.0654 282.3284 287.0536 288.0594 289.0564 289.1414 297.7313
3	Luteolin	12.823	C_15_H_10_O_6_	[M+H]^+^	287.055	287.055	0.000	10.5	121.0295 165.0182 258.0501 287.0556 288.0581 289.0616
4	Kaempferol-3-*O*-alpha-L-rhamnoside	11.791	C_21_H_20_O_10_	[M+H]^+^	433.113	433.114	0.001	11.5	129.0555 136.0649 287.0555 288.0582 289.0631 289.8441 320.8331 386.3335 399.8392 426.8164

**Figure 4:**
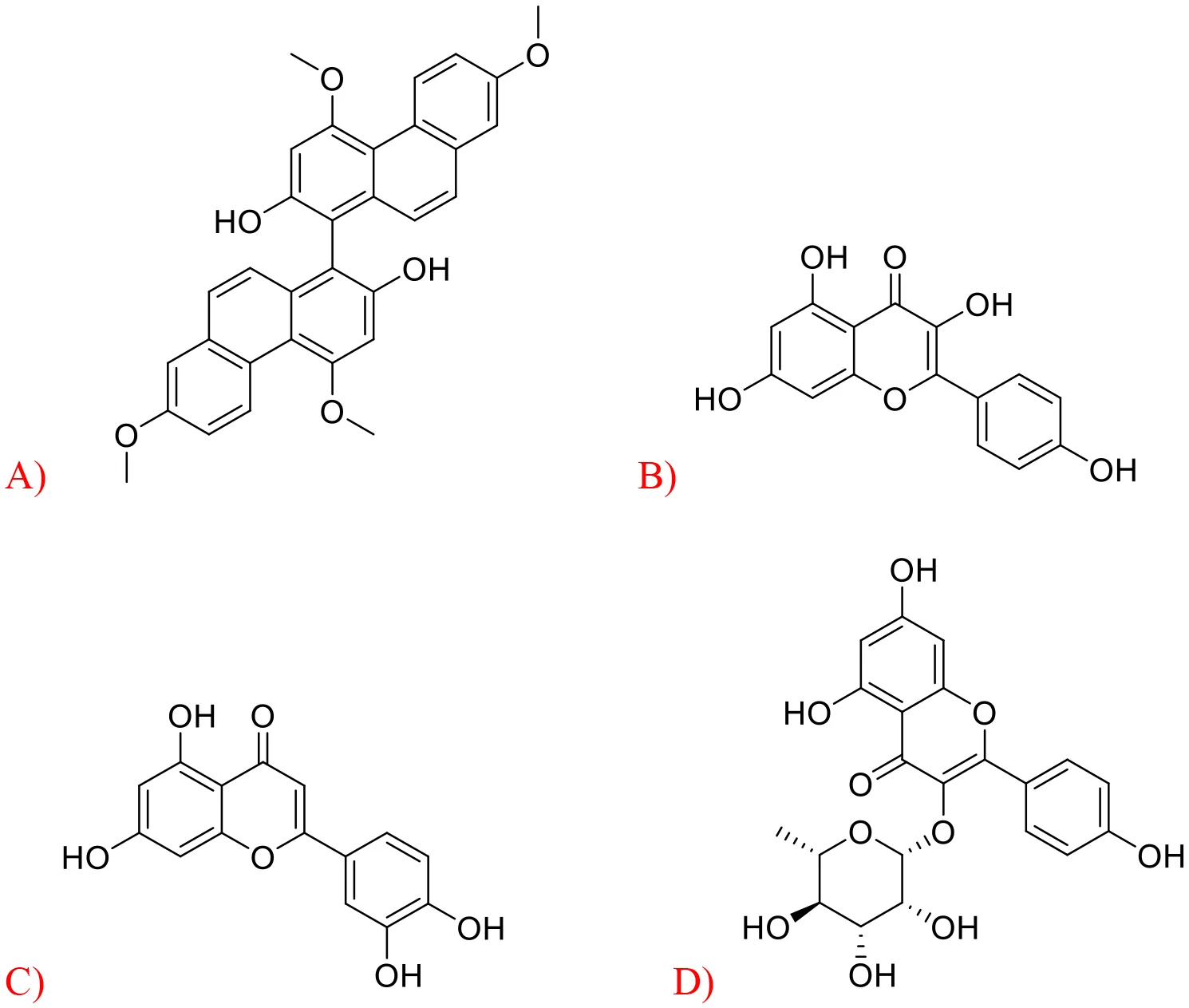
Structures of active compounds isolated from the leaves of *L. acidissima*: 2,2′-dihydroxy-4,7,4′,7′-tetramethoxy-1,1′-biphenanthrene **(A)**, kaempferol **(B)**, luteolin **(C)**, and kaempferol-3-*O*-α-L-rhamnopyranoside **(D)**.

Compound 1 (C1) was tentatively identified as 2,2′-dihydroxy-4,7,4′,7′-tetramethoxy-1,1′-biphenanthrene, with the molecular formula C_32_H_26_O_6_, a molecular weight of 506.6 g/mol, and an exact mass of 506.172939 g/mol. 2,2′-dihydroxy-4,7,4′,7′-tetramethoxy-1,1′-biphenanthrene was assigned based on the presence of a protonated molecular ion at m/z 507.181 ([M+H]^+^). The compound exhibited a retention time of 17.976 min and an accurate mass difference of +1.97 ppm between the measured and theoretical values, supporting the proposed elemental composition. The MS/MS spectrum showed characteristic fragment ions at m/z 332.808, 393.189, 395.201, and 396.207, which were considered to arise from fragmentation of the biphenanthrene core and loss of oxygenated substituents. Although several additional signals were detected in the spectrum, including dominant ions at m/z 506.243, 508.182, 511.296, and 579.087, these were present within a dense background region and lacked sufficient intensity or structural relevance; therefore, they were not considered for compound confirmation. The tentative identification was therefore based mainly on accurate mass measurement, retention behavior, and the characteristic fragmentation profile. The proposed structure was further supported by the ACD/Labs confidence score (>80%), indicating strong agreement between the experimental data and predicted spectral features. The mass spectral information for this compound is presented in **Table 1**; **Supplementary Figure 7**, and its structure is shown in **Figure 4A**. This is a biphenanthrene derivative in which two phenanthrene units with methoxy and hydroxy substituents are linked. This compound is reported here for the first time from *L. acidissima*, although it has previously been reported from *Cremastra appendiculata* (Xue et al., 2006). Several phenanthrene derivatives have also been reported from *Liparis nervosa* (Liu et al., 2024), *Bletilla striata* (Sui et al., 2021), and other plants.

Compound 2 (C2) was tentatively identified as kaempferol, with the molecular formula C_15_H_10_O_6_, a molecular weight of 286.24 g/mol, an exact mass of 286.047738 g/mol, and the IUPAC name of 3,5,7-trihydroxy-2-(4-hydroxyphenyl)-4H-1-benzopyran-4-one. Kaempferol was tentatively annotated based on its protonated molecular ion at *m/z* 287.054 ([M+H]^+^) together with its retention time of 12.848 min. The MS/MS spectrum exhibited a characteristic fragment ion at *m/z* 153.025, which is considered a diagnostic product ion of kaempferol. Additional product ions at *m/z* 258.051, 259.065 and 282.328 were observed, which correspond to neutral-loss fragmentation of the precursor ion and providing further support for the proposed structure. The precursor ion at *m/z* 287.054 was accompanied by isotopic peaks at *m/z* 288.059 and 289.056, while signals at *m/z* 289.141 and 297.731 were also detected with low abundance and are most likely attributable to naturally occurring isotopes, adduct ions, or background ions rather than true fragmentation products. The accurate mass measurement (mass error = -3.48 ppm), together with the characteristic fragmentation pattern and chromatographic retention time, enabled kaempferol to be distinguished from its structural isomer, luteolin, and tentatively annotated. In addition, the confidence level generated by ACD/Labs was above 80%, indicating strong agreement between the experimentally observed mass spectral features and the predicted fragmentation patterns, and thus providing additional support for the proposed structural assignment. The mass spectral information of this compound is given in **Table 1**; **Supplementary Figure 8**, and its structure is shown in **Figure 4B**. It is a tetrahydroxy flavonol with a characteristic flavonoid backbone. This compound has already been reported in *L. acidissima* (Khatun and Sen, 2024), supporting its identification. It has also been reported in many other plants, such as *Ginkgo biloba*, *Sophora japonica*, *Moringa oleifera*, and other medicinal and edible plants (Calderon-Montano et al., 2011).

Compound 3 (C3) was tentatively identified as luteolin with the molecular formula C_15_H_10_O_6_, a molecular weight of 286.24 g/mol, an exact mass of 286.047738 g/mol, and the IUPAC name of 2-(3,4-dihydroxyphenyl)-5,7-dihydroxy-4H-chromen-4-. Luteolin was tentatively annotated based on its protonated molecular ion at m/z 287.055 ([M+H]^+^) together with its retention time of 12.823 min. Although luteolin shares the same molecular formula and precursor ion as its structural isomer kaempferol, it was differentiated by its characteristic MS/MS fragmentation pattern. The spectrum exhibited diagnostic product ions at m/z 121.030 and 165.018, which are characteristic of Retro-Diels–Alder (RDA) cleavage of the flavone C-ring and are commonly reported for luteolin. An additional fragment ion at m/z 258.050 was observed, corresponding to neutral-loss fragmentation of the precursor ion and further supporting the proposed structure. The precursor ion at m/z 287.056 was accompanied by isotopic peaks at m/z 288.058 and 289.062, which are consistent with the natural isotopic distribution of the molecular ion. The excellent mass accuracy (mass error = 0.00 ppm), together with the diagnostic fragment ions and chromatographic retention time, enabled luteolin to be distinguished from its structural isomer, kaempferol, and tentatively annotated. The compound assignment was further supported by the ACD/Labs identification confidence score, which exceeded 80%, indicating a high level of agreement between the experimental data and the predicted spectral and structural information. The mass spectral information of this compound is given in **Table 1**; **Supplementary Figure 9**, and its structure is shown in **Figure 4C**. It is a naturally occurring tetrahydroxyflavone with a diphenyl-propane skeleton, which is characteristic of flavonoids, with four hydroxyl (-OH) groups. This compound has already been reported in *L. acidissima* (Dash et al., 2015), supporting our identification. It has also been reported in other plants, such as *Jatropha Podagrica* (Tran et al., 2025), *Lophatherum gracile* (Ding et al., 2024), and *Anthemis palestina* (Tabaza et al., 2024).

Compound 4 (C4) was tentatively identified as kaempferol-3-*O*-α-L-rhamnopyranoside or afzelin with the molecular formula C_21_H_20_O_10_, a molecular weight of 432.38 g/mol, an exact mass of 432.105647 g/mol, and IUPAC name of 5,7-dihydroxy-2-(4-hydroxyphenyl)-3-[(2R,3R,4R,5R,6S)-3,4,5-trihydroxy-6-methyloxan-2-yl]oxychromen-4-one. Kaempferol-3-*O*-α-L-rhamnoside was identified based on the precursor ion at m/z 433.113 ([M+H]^+^), with an accurate mass error of +2.31 ppm and a retention time of 11.791 min. The dominant MS/MS fragment at m/z 287.055 was assigned to the kaempferol aglycone formed by the cleavage of a rhamnose residue (loss of 146 Da), representing the key diagnostic ion for this compound. Additional fragments at m/z 129.056 and 136.065 further supported the characteristic fragmentation pattern of the kaempferol skeleton. The ions detected at m/z 288.058 and 289.063 were attributed to isotopic contributions of the aglycone fragment, whereas the minor higher-mass signals were considered non-diagnostic. The compound assignment was further supported by the ACD/Labs confidence score (>80%), indicating a high level of agreement between the experimental data and the predicted spectral and structural information. The structural assignment was further confirmed by proton nuclear magnetic resonance (¹H NMR) analysis, supporting the identification of kaempferol-3-*O*-α-L-rhamnoside. The mass spectral information of this compound is given in **Table 1**; **Supplementary Figure 10**, and its structure is shown in **Figure 4D**. The tentatively identified compound C4 was also confirmed using ^1^H-NMR spectroscopy (400 MHz) recorded in methanol-d4 (CD_3_OD). The ^1^H-NMR spectrum (MeOD, 400 MHz) of the compound (C4) revealed two signals at δ 6.19 (1H, d, J = 1.6 Hz) and δ 6.36 (1H, d, J = 1.6 Hz), which are well-known H-6, H-8, which are characteristic of the A-ring of kaempferol derivatives possessing a 5,7-dihydroxyl substitution pattern. Other two signals at δ 7.76 (2H, d, J = 8.4 Hz) 6.93 (2H, d, J = 8.4 Hz) formed an AA′BB′ spin system corresponding to H-2′/H-6′ and H-3′/H-5′, respectively, confirming the para-substituted B-ring typical of kaempferol. Similarly, the signals at δ 5.36 (d, overlapped), δ 0.91 (overlapped), were assigned to the α-L-rhamnopyranosyl moiety attached at C-3 of the aglycone. The anomeric proton resonance at δ 5.36 is consistent with an α-linked rhamnose residue, while the remaining resonances between δ 3.6 and 4.3 ppm arise from the sugar ring protons. The methyl doublet at approximately δ 0.91 is characteristic of the C-6 methyl group of rhamnose. The intense signal observed at approximately δ 4.8 ppm corresponds to the residual HOD peak originating from exchangeable deuterated methanol solvent and does not belong to the compound. Minor overlapping signals in the sugar proton region are expected because several rhamnose protons resonate within a narrow chemical shift range in CD_3_OD. Based on this data and by comparison with the literature (Akter et al., 2022), C4 was identified as kaempferol-3-*O*-alpha-L-rhamnoside. The 1H-NMR spectrum is shown in **Supplementary Figure 11**. It is a naturally occurring flavonoid glycoside reported in the Rutacea family and in other plant families, but has not yet been reported from *L. acidissima*; thus, this is its first report from this plant species. This compound has already been reported from *Pithecellobium dulce* (Akter et al., 2022), *Litsea glutinosa* (Bulbul et al., 2024), and other plants.

### 3.4 Antimicrobial, anthelmintic, and cytotoxicity activity of isolated compounds

**Figure 5:**
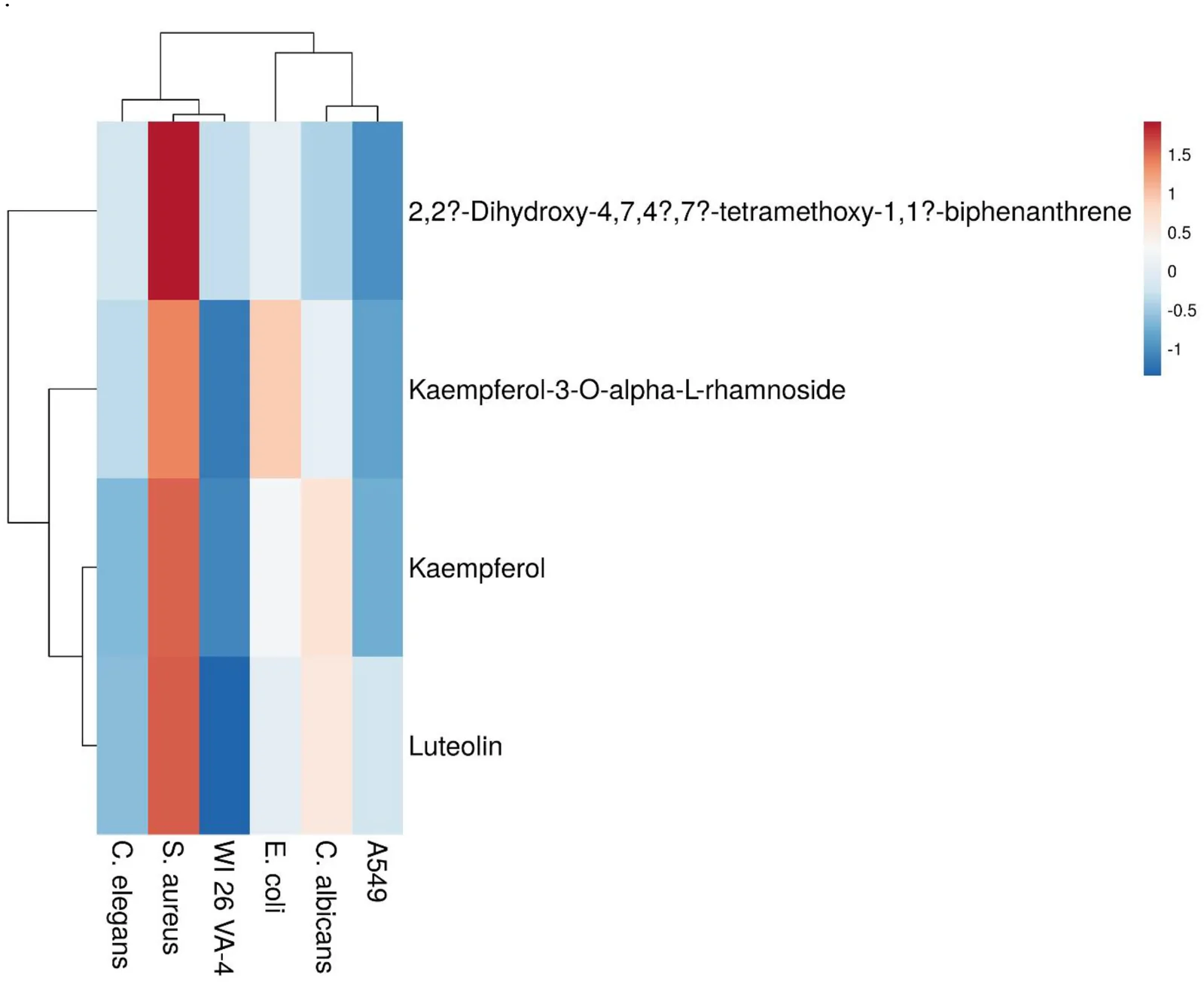
Heatmap of the antimicrobial, anthelmintic, and cytotoxic activities of compounds isolated from *L. acidissima*. The colour scale represents normalized activity values ranging from −1 to 1, with higher positive values indicating stronger activity and lower or negative values indicating weaker or no activity. The activity values were calculated and normalized as described in the Materials and Methods section.

The four active antimicrobial compounds isolated from *L. acidissima* were evaluated against bacterial (*S. aureus* and *E. coli*), fungal (*C. albicans*), helminthic (*C. elegans*), and mammalian cell models (A549 cancer cell lines and WI-26 VA4 normal cell lines). The percent inhibition and IC_50_ values for the antibacterial, antifungal, anthelmintic, and cytotoxic activities of the isolated compounds from *L. acidissima* are presented in **Supplementary Table 3**, while the overall bioactivities are presented as a heatmap in **Figure 5**.

C1 exhibited concentration-dependent activity against the test organisms and cell lines. The activity was significant against *S. aureus*, with a higher inhibition value (76% at 50 µg/mL) and an IC_50_ of 19 µg/mL, indicating high potency against Gram-positive bacteria. The activity was weaker against *E. coli*, with 39% inhibition at 50 µg/mL and an IC_50_ of 64 µg/mL, indicating limited efficacy against Gram-negative bacteria. The approximately three-fold higher IC_50_ against *E. coli* than *S. aureus* indicates a marked difference in susceptibility between the two bacterial species. The activity against *C. albicans* was moderate, with 30% inhibition at 20 µg/mL and an IC_50_ of 33 µg/mL. The compound also showed noticeable anthelmintic activity in the *C. elegans* model, with 34% inhibition at 5 µg/mL and an IC_50_ of 7 µg/mL. To our knowledge, the anthelmintic activity of this compound or closely related biphenanthrenes has not been previously reported. The cytotoxicity assessment of this compound revealed moderate activity against A549 cancer cells, with an inhibition value of 19% at 10 µg/mL and an IC_50_ of 35 µg/mL, indicating relatively low toxicity towards these cells. However, higher cytotoxicity was observed in WI-26 VA4 normal cell lines, with an inhibition value of 32% at 10 µg/mL and an IC_50_ of 16 µg/mL, indicating poor selectivity for cancer cells over normal cells. Given its antibacterial activity against *S. aureus* and anthelmintic activity, it warrants further study to better understand its therapeutic potential. However, its relatively higher cytotoxicity towards normal cell lines may need to be reduced to improve its safety.

There are no direct reports on the antibacterial, antifungal, and anthelmintic activities of 2,2′-dihydroxy-4,7,4′,7′-tetramethoxy-1,1′-biphenanthrene; however, structurally related biphenanthrene derivatives have been found to exhibit significant antimicrobial and cytotoxic activities. A biphenanthrene, 4, 8, 4′, 8′-Tetramethoxy (1, 1′-biphenanthrene)-2, 7, 2′, 7′-tetrol, isolated from the roots of *Bletilla striata*, shows significant effects against *S. aureus* and *B. subtilis* with an MIC of 8−16 µg/mL (Huang et al., 2021). In comparison, C1 showed an IC_50_ of 19 µg/mL against *S. aureus*. Although MIC and IC_50_ values represent different measures of antimicrobial activity and cannot be directly equated, the values fall within a similar concentration range, supporting the antibacterial potential of biphenanthrene-type compounds against Gram-positive bacteria. Similarly, several biphenanthrenes have shown promising effects against six Gram-positive bacteria, including *S. aureus* (Qian et al., 2015). The stronger activity of C1 against *S. aureus* than *E. coli* in the present study is consistent with these reports, which indicate that related biphenanthrenes may show greater activity against Gram-positive bacteria. Several phenanthrene dimers have also been reported to have significant antibacterial and antiproliferative effects (De Natale et al., 2022). Phenanthrene derivatives have also been reported to have broad-spectrum activity against phytopathogenic fungi (Zhou et al., 2016). The moderate activity of C1 against *C. albicans* (IC_50_: 33 µg/mL) is therefore consistent with the reported biological potential of related phenanthrene derivatives, although direct quantitative comparisons are limited by differences in fungal species and assay conditions. There are no reports of the anthelmintic activity of phenanthrene or biphenanthrene; our study reports it for the first time. The IC_50_ of 7 µg/mL observed against *C. elegans*, therefore, provides a potentially novel biological activity for this structural class. Two biphenanthrenes, namely bulbophythrins A and bulbophythrins B, exhibit significant cytotoxicity against some cancer cell lines (Xu et al., 2009). In contrast, C1 showed weaker activity against A549 cells (IC_50_: 35 µg/mL) but greater toxicity toward WI-26 VA4 normal cells (IC_50_: 16 µg/mL), indicating that its cytotoxic profile is not favorable for selective anticancer activity. Based on our reported bioactivities and the available literature, the newly isolated compound, C1, may be considered a promising candidate for further biological evaluation, particularly in view of its antibacterial and anthelmintic activities, although its cytotoxicity toward normal cells warrants further investigation.

C2 exhibited strong antibacterial activity against *S. aureus* with a significant inhibition of 85% at 50 µg/mL and an IC_50_ of 17 µg/mL. In comparison, its activity against *E. coli* was weaker, with a 50% inhibition at 50 µg/mL and an IC_50_ of 28 µg/mL, consistent with the reduced membrane permeability of Gram-negative bacteria. The approximately 1.6-fold higher IC_50_ against *E. coli* than *S. aureus* further indicates the greater susceptibility of the Gram-positive bacterium to C2. The activity against *C. albicans* was also significant, with 61% inhibition at 20 µg/mL and an IC_50_ of 10 µg/mL. The lower IC_50_ observed for *C. albicans* compared with both bacterial species indicates that C2 also has considerable antifungal activity under the present assay conditions. The anthelmintic activity in the *C. elegans* model is moderate, with a 26% inhibition at 5 µg/mL and an IC_50_ of 9 µg/mL. Cytotoxicity testing revealed that kaempferol is more toxic towards A549 cancer cell lines, with an inhibition of 24% at 10 µg/mL and an IC_50_ of 33 µg/mL, compared to WI-26 VA4 normal cell lines, where it showed an inhibition of 15% at 10 µg/mL and an IC_50_ of 42 µg/mL, indicating some selectivity towards cancer cells.

Kaempferol has shown significant antibacterial activity against several Gram-positive bacteria, including *S. aureus*, with an MIC value of 6-8 µg/mL (Wei et al., 2025). In the present study, kaempferol showed an IC_50_ of 17 µg/mL against *S. aureus*, indicating promising activity, although the potency was lower than the MIC range reported by Wei et al. (2025). However, these values should be compared with caution because MIC and IC_50_ represent different measures of antimicrobial activity and may be influenced by differences in assay conditions. However, the activity observed in the present study is in agreement with previous reports supporting kaempferol as an active antibacterial flavonoid. The stronger activity observed against *S. aureus* than *E. coli* in the present study is also consistent with previous reports describing greater susceptibility of Gram-positive bacteria to kaempferol and related flavonoids. The moderate antibacterial activity of kaempferol against *E. coli*, promising activity against a range of other Gram-positive and Gram-negative bacteria, and significant antifungal activity against *C. albicans* and other fungal species have been documented (for a review, see Periferakis et al., 2022). The IC_50_ of 10 µg/mL observed against *C. albicans* in the present study further supports the reported antifungal potential of kaempferol. This compound has also been found to reduce the antifungal resistance by inhibiting the expression of CDR1, CDR2, and MDR1 genes (Shao et al., 2016). There are no direct reports on the anthelmintic activity of kaempferol; however, its antiprotozoal potential against *E. histolytica*, *G. lamblia*, and other protozoans has been reported (Periferakis et al., 2022). Similarly, oral administration has been shown to exhibit significant anthelmintic activity against *Schistosoma mansoni* (Albuquerque et al., 2024). Thus, the moderate activity observed against *C. elegans* in the present study provides additional evidence of the broader antiparasitic potential of kaempferol, although direct comparisons should be made cautiously because of the differences in experimental models. The cytotoxicity and anticancer activities of kaempferol have been well documented across a range of cancer cell lines (de Morais et al., 2024). Londonkar (2016) also reported significant *in vitro* cytotoxicity of this compound against MCF-7 breast cancer and A549 lung cancer cell lines. The present A549 result (IC_50_: 33 µg/mL), together with the weaker effect on WI-26 VA4 cells (IC_50_: 42 µg/mL), is consistent with the previously reported cytotoxic potential of kaempferol while suggesting a modest degree of selectivity in the present assay. The significant antibacterial and anticancer activities, along with minimal effects on normal cells, suggest a favorable selectivity profile and warrant further biological evaluations and optimization for its potential therapeutic applications.

C3 showed significant antibacterial activity against *S. aureus*, with an inhibition value of 73% at 50 µg/mL and an IC_50_ of 19 µg/mL, confirming its high potency against Gram-positive bacteria. The activity against *E. coli* was lower, with 48% inhibition at 50 µg/mL and an IC_50_ of 44 µg/mL. This approximately two-fold difference in IC_50_ values indicates greater susceptibility of *S. aureus* than *E. coli* to C3. The antifungal activity of luteolin against *C. albicans* was reported as moderate, with 57% inhibition at 20 µg/mL and an IC_50_ of 16 µg/mL. The anthelmintic activity was also significant, with an inhibition value of 38% at 5 µg/mL and an IC_50_ of 8 µg/mL. To our knowledge, direct reports on the anthelmintic activity of luteolin against *C. elegans* are limited, making this finding of particular interest. Cytotoxicity assays revealed that luteolin is comparatively more cytotoxic towards A549 cancer cell lines with an inhibition value of 44% at 10 µg/mL and IC_50_ value of 9 µg/mL, while the normal cell lines WI-26 VA4 were found to be comparatively less sensitive with an inhibition value of 26% at 10 µg/mL and an IC_50_ value of 24 µg/mL. The approximately 2.7-fold higher IC_50_ value in WI-26 VA4 cells than in A549 cells further supports the relative selectivity of C3 toward the cancer cell line under the present experimental conditions.

The literature reports that luteolin is mostly active against Gram-positive bacteria and moderately active against Gram-negative bacteria. Luteolin has shown significant antibacterial activity against *S. aureus*, including methicillin-resistant *S. aureus* (MRSA), with growth inhibition at a MIC of 31.25 µg/mL. In comparison, the present study showed an IC_50_ of 19 µg/mL against *S. aureus*. Although MIC and IC_50_ values are different measures of antimicrobial activity and cannot be directly equated, the lower concentration associated with 50% inhibition in the present assay supports the strong antibacterial potential of luteolin against *S. aureus.* Luteolin has also been found to exhibit dose-dependent antibacterial activity against multidrug-resistant *E. coli* via biofilm inhibition and modulation of resistance gene expression (Ding et al., 2024). The IC_50_ of 44 µg/mL against *E. coli* in the present study indicates weaker activity than against *S. aureus*, consistent with the generally greater susceptibility of Gram-positive bacteria to luteolin. Selective antifungal activity of luteolin has been reported against Candida species, with an MIC of 37.5 µg/mL for *C. albicans* (Ivanov et al., 2020). The IC_50_ of 16 µg/mL observed against *C. albicans* in the present study indicates substantial antifungal activity, although direct comparison with the reported MIC should be interpreted cautiously because the endpoints and assay conditions differ. There are no direct reports of luteolin’s anthelmintic activity. However, antiprotozoal tests showed significant activity against *L. tropica* (MIC 12.5 µg/mL) and moderate activity against *A. castellanii* and *E. histolytica* (MIC 100 µg/mL), indicating its highest potential against *L. tropica* (Tileklioğlu and Aydın, 2025). The IC_50_ of 8 µg/mL observed against *C. elegans* therefore provides additional evidence of the antiparasitic potential of luteolin, although comparisons across different parasite models should be made cautiously. Reports on the cytotoxicity of luteolin suggest that it is more toxic to cancer cells than to normal cells. Luteolin has been reported to exhibit moderate activity against MDA-MB-231 cells (IC_50_ of 14.91 µM or 4.27 µg/mL), while being less cytotoxic towards MCF-7 cells (IC_50_ of 29.28 µM or 8.38 µg/mL) and exhibiting low cytotoxicity against normal fibroblasts (IC_50_ of 51.39 µM or 14.71 µg/mL). In the present study, luteolin showed an IC_50_ of 9 µg/mL against A549 cells compared with 24 µg/mL against WI-26 VA4 cells, giving a roughly 2.7-fold difference in sensitivity. Although the cell lines and experimental conditions differ from those reported previously, this pattern is consistent with the preferential cytotoxicity of luteolin toward cancer cells. These reports in the literature and the activities reported in this study suggest that luteolin may warrant further investigation for its antibacterial, antifungal, and anthelmintic potential.

C4 exhibited significant antibacterial activity against *S. aureus* with an inhibition value of 61% at 50 µg/mL and an IC_50_ of 22 µg/mL, confirming its high potency against Gram-positive bacteria. The activity against *E. coli* was modest, with 52% inhibition at 50 µg/mL and an IC_50_ of 35 µg/mL. The lower IC_50_ against *S. aureus* than *E. coli* indicates greater susceptibility of the Gram-positive bacterium to C4. The antifungal activity was moderate, with an inhibition value of 37% at 20 µg/mL and an IC_50_ of 32 µg/mL. The anthelmintic activity against *C. elegans* was also moderate, with an inhibition of 29% at 5 µg/mL and an IC_50_ of 10 µg/mL, consistent with the effects of flavonoid glycosides. To our knowledge, direct reports on the anthelmintic activity of purified afzelin are limited, making the present finding of interest. The cytotoxicity assays revealed that it inhibited A549 cancer cell lines with an inhibition value of 20% at 10 µg/mL and an IC_50_ value of 20 µg/mL, while showing lower toxicity towards WI-26 VA4 normal cell lines with an inhibition value of 14% at 10 µg/mL and an IC_50_ value of 33 µg/mL, indicating selectivity against tumor cell lines. The approximately 1.7-fold higher IC_50_ in WI-26 VA4 cells than A549 cells further supports the relative selectivity of C4 toward the cancer cell line under the present conditions.

According to published research, kaempferol-3-*O*-alpha-L-rhamnoside (afzelin) exhibits antibacterial and antifungal activities against *S. aureus*, *E. coli*, and *C. albicans*. Afzelin has shown antibacterial activity against *P. aeruginosa*, with an MIC of 31 µg/mL (Lee et al., 2014). In the present study, C4 showed IC_50_ values of 22 µg/mL against *S. aureus* and 35 µg/mL against *E. coli*. Although these IC_50_ values cannot be directly compared with the reported MIC because they represent different measures of antimicrobial activity, they indicate that C4 is active against both bacterial groups, with greater potency against *S. aureus*. Similarly, afzelin, when combined with silver nanoparticles, has been found to exhibit potent antibiofilm activity against *S. enterica* (Lotha et al., 2018). Afzelin has also shown potent antibacterial activity against *S. aureus*, *E. coli*, and *S. typhimurium* with an IC_50_ value of 0.125−0.25 mg/mL (Le et al., 2019). The IC_50_ values observed for C4 in the present study (22–35 µg/mL, equivalent to 0.022–0.035 mg/mL) are lower than the 0.125–0.25 mg/mL range reported by Le et al. (2019). However, this comparison should be interpreted cautiously because differences in assay systems, microbial strains, exposure conditions, and endpoint definitions can substantially influence IC_50_ values. The direct antifungal activity of afzelin has not been widely reported in the literature, although kaempferol rhamnosides have been reported with antifungal activities against *C. albicans* and other yeasts/fungal species (Tatsimo et al., 2012). The IC_50_ of 32 µg/mL observed against *C. albicans* in the present study therefore provides additional evidence for the antifungal potential of this compound class, although direct quantitative comparison is limited by the lack of comparable afzelin-specific data. The antiparasitic/anthelmintic reports on afzelin are also limited; however, afzelin-containing extracts have been found to possess significant anthelmintic effects against *Gliricidia sepium*, *Pithecellobium dulce*, and *Leucaena leucocephala* (Romero et al., 2020). The IC_50_ of 10 µg/mL observed against *C. elegans* provides further support for the antiparasitic potential associated with afzelin-containing preparations, although the use of crude extracts in previous studies prevents direct comparison with the purified compound tested here. The anticancer or cytotoxicity activities of afzelin are well documented. Afzelin has shown *in vivo* anti-tumor activity in a mouse carcinoma model, with 70% inhibition at 50 mg/kg (Akter et al., 2022). It has also been reported to have significant anticancer potential against gastric cancer cells by modulating apoptosis (Radziejewska et al., 2021). Consistent with these reports, C4 showed greater cytotoxicity toward A549 cells (IC_50_: 20 µg/mL) than WI-26 VA4 normal cells (IC_50_: 33 µg/mL), although the difference was relatively modest. These results and reports suggest the broad-spectrum potential of kaempferol-3-*O*-alpha-L-rhamnoside and support further study as a potential lead compound.

The differences in activity among C1-C4 also provide some preliminary structure–activity relationship (SAR) insights. C1, a biphenanthrene derivative containing two phenanthrene units with hydroxyl and methoxy substituents, showed antibacterial activity against *S. aureus* (IC_50_: 19 µg/mL), comparable to the flavonoids evaluated in this study. The activity of C1 is also consistent with previous reports showing that biphenanthrene derivatives can exhibit pronounced antibacterial activity against Gram-positive bacteria, including *S. aureus* (Huang et al., 2021; Qian et al., 2015). The relatively strong activity of C1 suggests that the extended aromatic biphenanthrene scaffold, together with its hydroxyl and methoxy groups, may contribute to antibacterial activity, potentially through interactions with bacterial membranes and other cellular targets. C1 also showed notable anthelmintic activity against *C. elegans* (IC50: 7 µg/mL), suggesting that the same structural features may contribute to activity beyond its antibacterial effects; however, the limited literature on purified biphenanthrenes prevents a clear structural interpretation of this activity However, because C1 represents a single biphenanthrene structure in the present study, the contribution of individual substituents to its activity cannot be clearly separated. Among the flavonoids, kaempferol (C2), which contains multiple hydroxyl groups on the flavonoid scaffold, showed the strongest activity against *S. aureus* (IC_50_: 17 µg/mL), followed by luteolin (C3; IC_50_:19 µg/mL) and the glycosylated derivative C4 (IC_50_: 22 µg/mL). The slightly lower activity of C4 compared with C2 may be related to the presence of the rhamnose moiety at the C-3 hydroxyl group. Glycosylation can alter the polarity, lipophilicity, and membrane interactions of flavonoids, and has been reported to influence their antibacterial activity (Górniak et al., 2019; Echeverría et al., 2017; Xie et al., 2015). The differences in anthelmintic activity among the flavonoids may also reflect structural variation: C3 showed the strongest activity (IC_50_: 8 µg/mL), followed by C2 (9 µg/mL) and the glycosylated C4 (10 µg/mL). The slightly lower activity of C4 may similarly suggest that glycosylation influences the interaction of flavonoid structures with biological targets, although this relationship cannot be established from the present data alone. Similarly, differences in the number and position of hydroxyl groups can affect the antibacterial activity of flavonoids by modifying their physicochemical properties and interactions with bacterial membranes and cellular targets (Górniak et al., 2019; Xie et al., 2015). Structural differences were also reflected in the cytotoxicity profiles. C3 showed the strongest activity against A549 cancer cells (IC_50_: 9 µg/mL), whereas C2 and C4 showed weaker effects (IC_50_: 33 and 20 µg/mL, respectively). C1, in contrast, showed greater toxicity toward WI-26 VA4 normal cells (IC_50_: 16 µg/mL) than A549 cells (IC_50_: 35 µg/mL), indicating that its structural features did not confer the same degree of cancer-cell selectivity observed for the flavonoids. These differences suggest that hydroxylation, glycosylation, and the overall flavonoid scaffold may influence cytotoxic activity and selectivity, but further testing with structurally related compounds would be required to establish a reliable SAR. However, these observations should be regarded as preliminary because C1-C4 belong to different structural classes and differ in several structural features. Therefore, the present data cannot establish a definitive SAR, but they provide useful indications for future studies involving a larger series of structurally related compounds.

The greater susceptibility of the Gram-positive *S. aureus* compared with the Gram-negative *E. coli* observed for all four compounds may be related to differences in their cell-envelope structures. Gram-negative bacteria possess an additional outer membrane containing lipopolysaccharide (LPS), which forms an important permeability barrier and can limit the entry and accumulation of many antimicrobial compounds. In contrast, Gram-positive bacteria lack this outer membrane, which may allow some phytochemicals to interact more readily with the cytoplasmic membrane or other cellular targets (Maher and Hassan, 2023; Delcour, 2009). Flavonoids can act through several mechanisms, including disruption of membrane integrity, alteration of membrane permeability, interference with energy metabolism and respiratory processes, inhibition of nucleic acid synthesis, and modulation of efflux pumps (Abou Baker, 2022; Cushnie and Lamb, 2005). The lipophilicity and structural features of flavonoids may also influence their ability to interact with bacterial membranes and contribute to differences in activity among bacterial species (Yuan et al., 2021). Thus, the stronger activity observed against *S. aureus* may partly reflect easier access to cellular targets in the absence of the additional Gram-negative outer-membrane barrier. However, differences in bacterial susceptibility can involve several factors, including membrane composition, permeability, efflux mechanisms, and compound-specific interactions, and the precise mechanisms responsible for the activity of C1-C4 remain to be experimentally established.

The antimicrobial, anthelmintic, and cytotoxic activities observed in the isolated compounds reveal previously unexplored pharmacological potential of *L. acidissima* leaf constituents beyond their traditional use. Overall, compounds C1, C3, and C4 are reported here for the first time from this plant, and several of the biological activities demonstrated in this study have not been previously described for these compounds. The observed bioactivities across different biological systems highlight these compounds as promising candidates for further pharmacological investigation. In addition, the varying responses against Gram-positive and Gram-negative bacteria suggest possible selective mechanisms of action, while the differences in activity observed among C1-C4 indicate that their distinct structural features may contribute to differences in antimicrobial potency, warranting further mechanistic and structure-activity relationship studies.

### 3.5 ADME evaluation

ADME properties were evaluated to determine whether or not a chemical agent exhibits drug-like behavior. As given in **Table 2**, the components exhibit a favorable pharmacokinetic profile.

**Table 2:** Assessment of ADME scores conducted for *Limonia acidissima* identified compounds.

No.	Compounds	Solute molecular weight^1^	QPlogHERG^2^	QPPCaco^3^ (nm/s)	QPPMDCK^4^ (nm/s)	Rule of five^5^	Rule of three^6^
1	2,2′-dihydroxy-4,7,4′,7′-tetramethoxy-1,1′-biphenanthrene (C1)	506.554	-6.725	1832.109	951.852	2	2
2	Kaempferol (C2)	286.24	-5.201	51.24	19.934	0	0
3	Luteolin (C3)	286.24	-5.023	40.856	15.606	0	0
4	Kaempferol 3-*O-*α-L- rhamnopyranoside (C4)	432.383	-5.517	16.23	5.753	1	1

^1^130–725, ^2^(concern below − 5), ^3^a < 25 is poor and a > 500 is great, ^4^a < 25 is poor and a > 500 is great, ^5^Maximum is 4, ^6^Maximum is 3.

and do not seem to be in violation of the Lipinski rule (except kaempferol (C2) and Luteolin (C3)). In addition, they do not possess any qualities that are mutagenic or carcinogenic. Based on these results, it is clear that these ligands have the potential to be developed into drugs.

#### 3.5.1 *In silico* approach and molecular docking analysis

For the purpose of performing molecular docking between the target protein and the ligands, the Glide module was used (Ghalla et al., 2018; Lalit et al., 2013). It was determined that a significant docking score was achieved by the interaction of the top ligands with the amino acids found in the target protein. **Supplementary Table 2** displays the docking scores for the ligands in relation to different receptors.

The 2D binding interactions of the identified ligands from *L. acidissima* leaves, 2,2′-dihydroxy-4,7,4′,7′-tetramethoxy-1,1′-biphenanthrene (C1), kaempferol (C2), luteolin (C3), and kaempferol 3-*O*-α-L-rhamnopyranoside (C4) with their respective receptors were analyzed.

**Figure 6:**
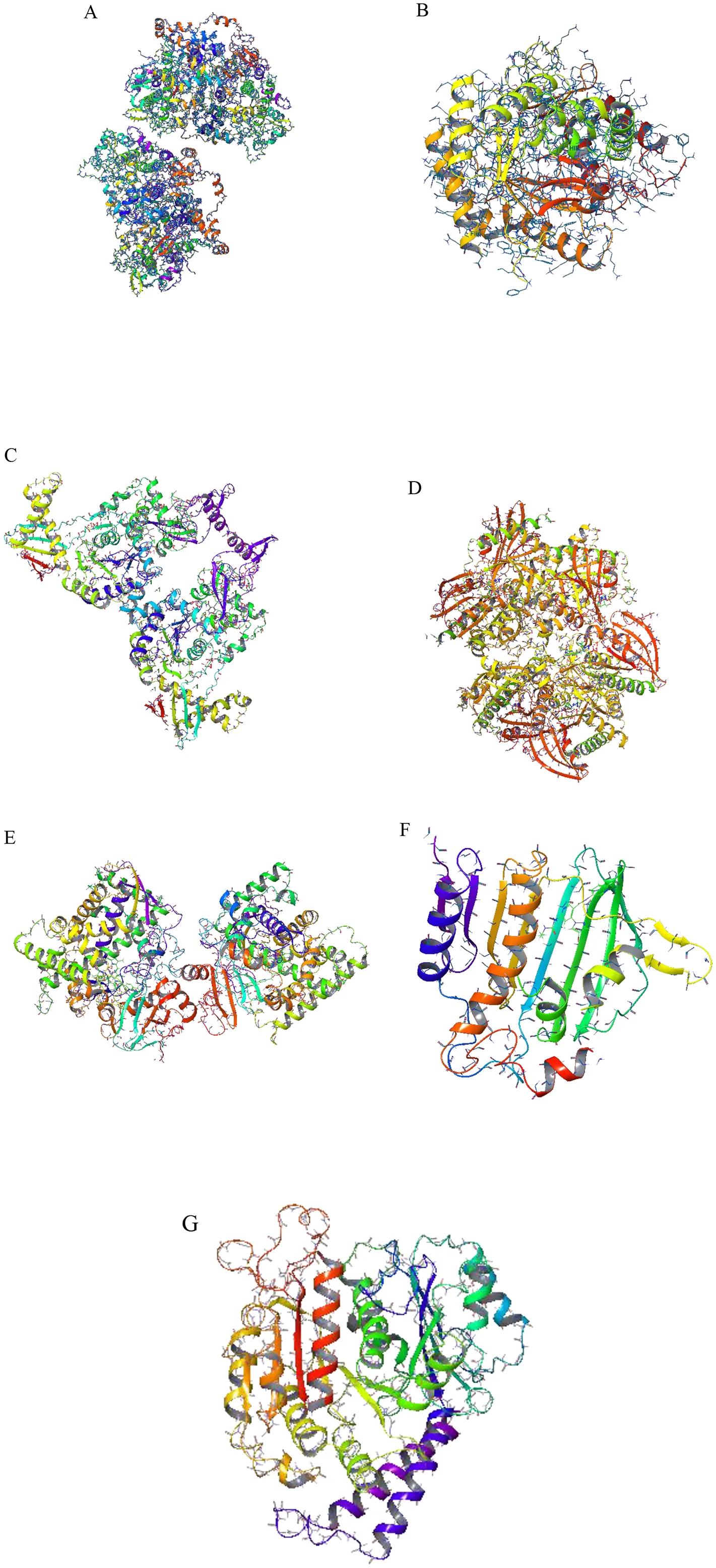
Optimized and 3D versions of receptors of **(A)** 1CX2, **(B)** 1TV5, **(C)** 2Y2H, **(D)** 3PHC, **(E)** 5TZ1, and **(F)** 6F86, and 6E88 **(G)**.

The receptors considered in this study included human COX-2 (PDB ID: 1CX2), *P. falciparum* DHFR (1TV5), *S. aureus* PBP-1b (2Y2H), *P. falciparum* purine nucleoside phosphorylase (3PHC), *C. albicans* CYP51 (5TZ1), *C. elegans* GDP-microtubule (6E88), and *E. coli* PBP (6F86). The COX-2 docking results were compared with the cytotoxicity data obtained using the A549 cell line, whereas 3PHC was included as an exploratory target to evaluate broader ligand–protein interactions. The detailed molecular docking results are presented in **Figures 6A–G**.

The molecular docking analysis revealed distinct binding behaviors for the four investigated compounds, namely kaempferol-3-*O*-α-L-rhamnoside, luteolin, kaempferol, and 2,2′-dihydroxy-4,7,4′,7′-tetramethoxy-1,1′-biphenanthrene, against different target proteins. For the human COX-2 protein (PDB ID: 1CX2), selected as the molecular target for comparison with the cytotoxicity results obtained in the A549 lung cancer cell line, kaempferol-3-*O*-α-L-rhamnoside exhibited the strongest binding affinity with a docking score of -7.751, forming a hydrogen bond with Tyr385 and interacting with several amino acid residues within the active site. Luteolin and kaempferol also demonstrated favorable binding, although with slightly lower affinities, whereas 2,2′-dihydroxy-4,7,4′,7′-tetramethoxy-1,1′-biphenanthrene showed an almost negligible docking score, indicating a very weak interaction with the receptor. These findings suggest that the rhamnose moiety attached to the kaempferol skeleton may enhance ligand–receptor interactions and improve binding stability.

For the 1TV5 protein from *Pf*-DHFR, kaempferol-3-*O*-α-L-rhamnoside again displayed the highest binding affinity, with a docking score of -9.375. This compound formed two hydrogen bonds and one π–π interaction with Tyr528, indicating strong stabilization within the binding pocket. Kaempferol and luteolin also showed favorable docking scores of -8.625 and -8.413, respectively, and both established π–π interactions with Tyr528. In contrast, 2,2′-dihydroxy-4,7,4′,7′-tetramethoxy-1,1′-biphenanthrene failed to exhibit any significant interaction with this receptor, suggesting poor compatibility with the active site.

A different trend was observed for the 2Y2H protein from *S. aureus*. In this case, kaempferol demonstrated the strongest binding affinity with a docking score of -5.994, supported by one hydrogen bond with Met556 and two π-π interactions with Tyr498. Luteolin exhibited a very similar binding pattern and only slightly lower affinity. Although kaempferol-3-*O*-α-L-rhamnoside formed four hydrogen bonds, its overall docking score was less favorable, indicating that the number of hydrogen bonds alone does not determine binding strength and that molecular orientation within the binding pocket is equally important.

For the *Plasmodium falciparum* purine nucleoside phosphorylase (PDB ID: 3PHC), included as an exploratory target, the three flavonoids exhibited comparable docking performances. Luteolin achieved the best docking score (-7.913), followed closely by kaempferol-3-*O*-α-L-rhamnoside (-7.784) and kaempferol (-7.752). Notably, kaempferol-3-*O*-α-L-rhamnoside formed the highest number of hydrogen bonds (five) along with a π–π interaction involving Tyr160, suggesting a stable ligand-protein complex despite only a marginal difference in binding energy compared with the other flavonoids.

The strongest overall docking results were observed for the 5TZ1 protein from *C. albicans*. Luteolin exhibited the highest binding affinity with a docking score of -9.655, followed by kaempferol-3-*O*-α-L-rhamnoside (-9.387) and kaempferol (-9.308). Interestingly, 2,2′-dihydroxy-4,7,4′,7′-tetramethoxy-1,1′-biphenanthrene, which generally performed poorly against most receptors, displayed a relatively favorable docking score of -7.743 in this target and formed two hydrogen bonds as well as two π–π interactions. This observation suggests a possible selective antifungal potential for 2,2′-dihydroxy-4,7,4′,7′-tetramethoxy-1,1′-biphenanthrene against *C. albicans*.

In the case of the 6F86 protein from *E. coli*, luteolin again showed the strongest binding affinity with a docking score of -6.367, primarily through the formation of a hydrogen bond with Asp73 kaempferol and kaempferol-3-*O*-α-L-rhamnoside also interacted with the receptor but with lower binding affinities. Consistent with previous observations, 2,2′-dihydroxy-4,7,4′,7′-tetramethoxy-1,1′-biphenanthrene exhibited the weakest interaction, indicating limited activity toward this bacterial target.

Finally, for the 6E88 receptor, kaempferol-3-*O*-α-L-rhamnoside once again emerged as the most potent ligand, displaying a docking score of -9.274 and forming four hydrogen bonds that contributed to a highly stable protein–ligand complex. Luteolin and kaempferol also demonstrated strong binding affinities but were less favorable than kaempferol-3-O-α-L-rhamnoside. Although 2,2′-dihydroxy-4,7,4′,7′-tetramethoxy-1,1′-biphenanthrene formed two hydrogen bonds and one π–π interaction, its docking score remained substantially weaker, placing it last among the tested compounds.

When the docking results are considered alongside the experimental bioactivity data, several trends become apparent. The strongest correspondence was observed for *S. aureus*, where kaempferol (C2) showed the highest experimental antibacterial potency (IC_50_: 17 µg/mL) and also produced the most favorable docking score among the tested compounds against the corresponding target (−5.994). Luteolin (C3) also showed strong experimental activity against *S. aureus* (IC_50_: 19 µg/mL) and favorable interactions with the bacterial target. Similarly, luteolin showed the strongest docking score against the *E. coli* target (−6.367), consistent with its comparatively favorable, although weaker, experimental activity against *E. coli* (IC_50_: 44 µg/mL). These observations provide supportive evidence that the predicted interactions may contribute to the observed biological effects. The molecular docking analysis complements the experimental bioassays by providing a structural perspective on the possible interactions between the isolated compounds and biologically relevant protein targets. Although docking cannot establish the exact mechanism of action, it helps identify plausible ligand–target interactions and may assist in prioritizing compounds for future mechanistic investigations. The overall agreement observed for several antibacterial targets, particularly *S. aureus* and *E. coli*, supports the relevance of the selected molecular targets. However, differences between docking predictions and experimental activities emphasize the complexity of biological responses and highlight the need for further experimental validation. However, the correspondence was not universal across all compounds and targets, indicating that docking results should be interpreted as supportive rather than definitive evidence of biological activity.

The lack of a strict quantitative relationship between docking scores and experimental activity is not unexpected. Molecular docking primarily estimates the favorability of ligand–receptor interactions within a predefined binding site and does not fully account for factors that determine cellular or organism-level activity, including membrane permeability, compound uptake, efflux, metabolic transformation, protein flexibility, solvent effects, target abundance, and possible differences between the selected computational target and the actual biological target responsible for the phenotype. In addition, docking scoring functions are simplified approximations of binding free energy and may not reliably reproduce experimental potency rankings. Thus, the strong docking scores observed for C4 and other compounds should be interpreted as evidence of plausible target interactions rather than direct predictions of their *in vitro* potency (Warren et al., 2006; Pagadala et al., 2017). Overall, the docking analysis provides useful mechanistic hypotheses that can guide future target-validation studies and support the rational prioritization of lead compounds.

### 3.6 Study limitations and future perspectives

Despite the promising antimicrobial, anthelmintic, cytotoxic, and *in silico* findings, several limitations should be acknowledged. The biological evaluation was performed using a limited number of microbial and cellular models, and the antimicrobial mechanisms of the isolated compounds were not experimentally investigated. In addition, molecular docking provides predictive evidence of possible ligand–target interactions but does not establish target engagement or mechanism of action in biological systems. The cytotoxicity assessment was also limited to one cancer cell line and one normal cell line, while the anthelmintic evaluation was performed using *C. elegans*. Future studies should therefore investigate a broader panel of clinically relevant and drug-resistant pathogens, establish minimum inhibitory concentrations and time-dependent killing profiles, and examine mechanisms of action using membrane-permeability assays, enzyme inhibition studies, transcriptomic or proteomic approaches, and target-validation experiments. Further *in vivo* studies, pharmacokinetic evaluation, and toxicity profiling will also be required to determine whether the most promising compounds, particularly C2 and C3, can progress toward therapeutic development.

## 4 Conclusion

The present study provide support for the traditional use of *L. acidissima* and suggests its broad pharmacological potential. The bioassay-guided fractionation of the acetone extract of *L. acidissima* yielded four tentatively identified compounds, all of which exhibited notable antimicrobial activity against Gram-positive bacteria, with limited activity against Gram-negative bacteria and yeast. The compounds also showed moderate anthelmintic activity and selective cytotoxicity effects. Notably, compounds C1, C3, and C4 may be reported for the first time from this plant, and several of their associated biological activities are reported for the first time in this study. The docking results suggest that kaempferol-3-*O*-α-L-rhamnoside possesses the most consistent and strongest binding profile across the majority of investigated targets, largely due to its ability to establish multiple stabilizing interactions within the active sites. Luteolin also demonstrated remarkable binding affinity, particularly against fungal and bacterial targets, and achieved the best docking scores for several receptors. Kaempferol showed moderate to strong activity and was notably the most effective compound against the *S. aureus* target. In contrast, 2,2′-dihydroxy-4,7,4′,7′-tetramethoxy-1,1′-biphenanthrene generally exhibited poor binding performance, with the exception of its potentially favorable interaction with the *C. albicans* receptor. Taken together, these findings suggest that kaempferol-3-*O*-α-L-rhamnoside and luteolin are the most promising candidates for further *in vitro* and *in vivo* evaluation aimed at further assessing their therapeutic potential. The findings highlight the significance of bioassay-guided isolation and *in silico* analysis as useful approaches in drug discovery, providing a robust foundation for future investigations into their mechanisms of action and therapeutic potential. These efforts may ultimately contribute to the identification of potential therapeutic agents.

## Data Availability

The datasets presented in this study can be found in online repositories. The names of the repository/repositories and accession number(s) can be found in the article/**Supplementary Material**.

## References

[B1] Abou Baker D. H. (2022). An ethnopharmacological review on the therapeutic properties of flavonoids and their mechanisms of actions: A comprehensive review based on up to date knowledge. Toxicol. Rep. 9, 445–469. doi: 10.1016/j.toxrep.2022.03.011 35340621 PMC8943219

[B2] Ahsan R. Paul S. Alam M. S. Rahman A. M. (2025). Synthesis, biological properties, in silico ADME, molecular docking studies, and FMO analysis of chalcone derivatives as promising antioxidant and antimicrobial agents. ACS Omega 10, 4367–4387. doi: 10.1021/acsomega.4c06897 39959036 PMC11822702

[B3] Akter M. Parvin M. S. Hasan M. M. Rahman M. A. A. Islam M. E. (2022). Anti-tumour and antioxidant activity of kaempferol-3-O-alpha-L-rhamnoside (Afzelin) isolated from Pithecellobium dulce leaves. BMC Complementary Med. Ther. 22, 169. doi: 10.1186/s12906-022-03633-x 35733130 PMC9219166

[B4] Albuquerque M. Luz R. Rodrigues V. Roquini D. Umehara E. de Moraes J. . (2024). Oral administration of kaempferol isolated from Baccharis mattogrosensis enables *In vivo* activity against Schistosoma mansoni. Chem. Biodivers. 21, e202401452. doi: 10.1002/cbdv.202401452 39136606

[B5] Ali A. Abuzer A. Musarrat H. W. Mohammad A. R. Mohamed J. A. Faizul A. (2022). An insight into the structural requirements and pharmacophore identification of carbonic anhydrase inhibitors to combat oxidative stress at high altitudes: An in-silico approach. Curr. Issues Mol. Biol. 44, 1027–1045. doi: 10.3390/cimb44030068 35723291 PMC8947748

[B6] Angelini P. (2024). Plant-derived antimicrobials and their crucial role in combating antimicrobial resistance. Antibiotics 13, 746. doi: 10.3390/antibiotics13080746 39200046 PMC11350763

[B7] Assefa T. Tesso H. Ramachandran V. P. Guta L. Demissie T. B. Ombito J. O. . (2023). In silico molecular docking analysis, cytotoxicity, and antibacterial activities of constituents of fruits of Cucumis dipsaceus. ACS Omega 9, 1945–1955. doi: 10.1021/acsomega.3c08866 38222496 PMC10785779

[B8] Banerjee S. Singha S. Laskar S. Chandra G. (2011). Efficacy of Limonia acidissima L. (Rutaceae) leaf extract on larval immatures of Culex quinquefasciatus Say 1823. Asian Pacific J. Trop. Med. 4, 711–716. doi: 10.1016/s1995-7645(11)60179-x 21967694

[B9] Bhat H. R. Singh U. P. Gahtori P. Ghosh S. K. Gogoi K. Prakash A. . (2015). Synthesis, docking, *In vitro* and *In vivo* antimalarial activity of hybrid 4-aminoquinolineâ “1,3,5-triazine derivatives against wild and mutant malaria parasites. Chem. Biol. Drug Des. 86, 265–271. doi: 10.1111/cbdd.12490 25487527

[B10] Bhole R. P. Bonde C. G. Bonde S. C. Chikhale R. V. Wavhale R. D. (2021). Pharmacophore model and atom-based 3D quantitative structure activity relationship (QSAR) of human immunodeficiency virus-1 (HIV-1) capsid assembly inhibitors. J. Biomol. Struct. Dyn. 39, 718–727. doi: 10.1080/07391102.2020.1715258 31928140

[B11] Bulbul I. Hossain M. Haque M. R. Al-Mansur M. A. Hasan C. M. Al Hasan A. . (2024). Two rare flavonoid glycosides from Litsea glutinosa (Lour.) CB Rob.: Experimental and computational approaches endorse antidiabetic potentiality. BMC Complementary Med. Ther. 24, 69. doi: 10.1186/s12906-024-04337-0 38302935 PMC10832099

[B12] Calderón-Montano M. J. Burgos-Morón E. Pérez-Guerrero C. López-Lázaro M. (2011). A review on the dietary flavonoid kaempferol. Mini-Rev. Med. Chem. 11, 298–344. doi: 10.2174/138955711795305335 21428901

[B13] Cédric Y. Yaghoobi M. Nadia N. A. C. Besati M. Che S. T. Sidiki N. N. A. . (2024). *In vitro* anthelmintic activity of ethanol and aqueous extracts of Terminalia macroptera and Bridelia micrantha against Heligmosomoides polygyrus, Caenorhabditis elegans and in-silico molecular docking evaluation of some isolated phytoconstituents. S. Afr. J. Bot. 164, 356–365. doi: 10.1016/j.sajb.2023.11.053

[B14] Christalin B. Besati M. Christelle Nadia N. A. Yaghoobi M. Cédric Y. Ciancia C. . (2024). *In vitro* anthelmintic activities of Khaya anthotheca and Faidherbia albida extracts used in Chad by traditional healers for the treatment of helminthiasis and in silico study of phytoconstituents. J. Trop. Med. 2024, 8564163. doi: 10.1155/2024/8564163 PMC11226339 38974476

[B15] Cushnie T. T. Lamb A. J. (2005). Antimicrobial activity of flavonoids. Int. J. Antimicrob. Agents 26, 343–356. doi: 10.1016/j.ijantimicag.2005.09.002 16323269 PMC7127073

[B16] Daphedar A. B. Sanjay S. Majani P. J. Kaddipudi R. B. Hujaratti S. B. Kakkalmeli A. A. . (2024). Evaluation of antioxidant and antibacterial activities of silver nanoparticles derived from Limonia acidissima L. fruit extract. Curr. Res. Green Sustain. Chem. 8, 100399. doi: 10.1016/j.crgsc.2024.10039942574925

[B17] Dash J. R. Pradhan D. Tripathy G. Behera B. (2015). Determination of some isoflavonoids and flavonoids from Limonia acidissima L. by HPLC-UV. Pharmacol. Online 3, 116–122.

[B18] Delcour A. H. (2009). Outer membrane permeability and antibiotic resistance. Biochim. Biophys. Acta (BBA)-Proteins Proteomics 1794, 808–816. doi: 10.1016/j.bbapap.2008.11.005 19100346 PMC2696358

[B19] de Morais E. F. de Oliveira L. Q. R. Farias Morais H. G. D. Souto Medeiros M. R. D. Freitas R. D. A. Rodini C. O. . (2024). The anticancer potential of kaempferol: A systematic review based on *in vitro* studies. Cancers 16, 585. doi: 10.3390/cancers16030585 38339336 PMC10854650

[B20] De Natale A. Pollio A. De Marco A. Luongo G. Di Fabio G. Zarrelli A. (2022). Phenanthrene dimers: Promising source of biologically active molecules. Curr. Top. Med. Chem. 22, 939–956. doi: 10.2174/1568026621666210813113918 34392822

[B21] Ding Y. Wen G. Wei X. Zhou H. Li C. Luo Z. . (2024). Antibacterial activity and mechanism of luteolin isolated from Lophatherum gracile Brongn. against multidrug-resistant Escherichia coli. Front. Pharmacol. 15, 1430564. doi: 10.3389/fphar.2024.1430564 38983919 PMC11232434

[B22] Dogbey B. F. Ibrahim S. Abobe J. A. E. (2020). Comparison of antioxidant and antimicrobial activities of acetone and water extracts of Theobroma cacao beans. Adv. Microbiol. 10, 478. doi: 10.4236/aim.2020.109036

[B23] Echeverría J. Opazo J. Mendoza L. Urzúa A. Wilkens M. (2017). Structure-activity and lipophilicity relationships of selected antibacterial natural flavones and flavanones of Chilean flora. Molecules 22, 608. doi: 10.3390/molecules22040608 PMC6154607 28394271

[B24] Fogel G. B. Cheung M. Pittman E. Hecht D. (2008). In silico screening against wild-type and mutant Plasmodium falciparum dihydrofolate reductase. J. Mol. Graphics Modell. 26, 1145–1152. doi: 10.1016/j.jmgm.2007.10.006 18037315

[B25] Friesner R. A. Banks J. L. Murphy R. B. Halgren T. A. Klicic J. J. Mainz D. T. . (2004). Glide: A new approach for rapid, accurate docking and scoring. 1. Method and assessment of docking accuracy. J. Med. Chem. 47, 1739–1749. doi: 10.1021/jm0306430 15027865

[B26] Ghalla H. Noureddine I. Fehmi B. Ahmet A. (2018). Intermolecular interactions and molecular docking investigations on 4-methoxybenzaldehyde. Comput. Mater. Sci. 149, 291–300. doi: 10.1016/j.commatsci.2018.03.04242574925

[B27] Górniak I. Bartoszewski R. Króliczewski J. (2019). Comprehensive review of antimicrobial activities of plant flavonoids. Phytochem. Rev. 18, 241–272. doi: 10.1007/s11101-018-9591-z

[B28] Haraguchi A. Harris B. Feasby N. Thekkiniath J. (2024). Prevalence of anti-parasitic drug resistance in various areas of the world. Indian J. Veterinary Public Health| Volume 10, 1. doi: 10.62418/ijvph.10.1.2024.1-11

[B29] Harshali A. L. Ekata M. Nikube P. Borse L. B. Borse S. L. S. P. (2015). Antibacterial activity of Limonia acidissima. Pharma Sci. Monitor 6, 12–24.

[B30] Hu H. Hu C. Peng J. Ghosh A. K. Khan A. Sun D. . (2021). Bioassay-guided interpretation of antimicrobial compounds in Kumu, a TCM preparation from Picrasma quassioides’ stem via UHPLC-Orbitrap-ion trap mass spectrometry combined with fragmentation and retention time calculation. Front. Pharmacol. 12, 761751. doi: 10.3389/fphar.2021.761751 34776978 PMC8581800

[B31] Hu H. Tekin V. Hu B. Yaghoobi M. Khan A. Ghosh A. K. . (2023). Metabolic profiling of Chimonanthus grammatus via UHPLC-HRMS-MS with computer-assisted structure elucidation and its antimicrobial activity. Front. Plant Sci. 14, 1138913. doi: 10.3389/fpls.2023.1138913 37229132 PMC10205022

[B32] Huang X. J. Xiong N. Chen B. C. Luo F. Huang M. Ding Z. S. . (2021). The antibacterial properties of 4, 8, 4′, 8′-tetramethoxy (1, 1′-biphenanthrene)-2, 7, 2′, 7′-tetrol from fibrous roots of Bletilla striata. Indian J. Microbiol. 61, 195–202. doi: 10.1007/s12088-021-00932-8 PMC8039075 33927460

[B33] Ilango K. Chitra V. (2010). Wound healing and anti-oxidant activities of the fruit pulp of Limonia acidissima Linn (Rutaceae) in rats. Trop. J. Pharm. Res. June 9, 223–230. doi: 10.4314/tjpr.v9i3.56281

[B34] Intekhab J. Aslam M. (2009). Isolation of a flavonoid from Feronia limonia. J. Saudi Chem. Soc 13, 295–298. doi: 10.1016/j.jscs.2009.10.01242574925

[B35] Işık A. Acar Ç.U. Karayel A. Ahmad I. Patel H. Çelik İ. . (2024). Synthesis, DFT calculations, in silico studies, and antimicrobial evaluation of benzimidazole-thiadiazole derivatives. ACS Omega 9, 18469. doi: 10.1021/acsomega.4c00543 PMC11044166 38680334

[B36] Ivanov M. Kannan A. Stojković D. S. Glamočlija J. Calhelha R. C. Ferreira I. C. . (2020). Flavones, flavonols, and glycosylated derivatives—Impact on Candida albicans growth and virulence, expression of cdr1 and erg11, cytotoxicity. Pharmaceuticals 14, 27. doi: 10.3390/ph14010027 33396973 PMC7824033

[B37] Jamil A. Rofida S. Priyani D. Nabila W. Wulandari E. (2019). “ Phytochemical screening and antimicrobial activity of Limonia acidissima ethanol extract against microbes from clinical isolates”, in: Proceedings of the 2nd Health Science International Conference (HSIC 2019) Setúbal, Portugal: SCITEPRESS – Science and Technology Publications, Lda., 152–156.

[B38] Jamshidi-Kia F. Lorigooini Z. Amini-Khoei H. (2018). Medicinal plants: Past history and future perspective. J. Herbmed Pharmacol. 7, 1–7. doi: 10.15171/jhp.2018.01

[B39] Khan A. Panda S. K. Hu H. Schoofs L. Luyten W. (2025a). Ethnomedicinal documentation, phytochemical characterization, and biological evaluation of the traditional medicinal plants from Swat region of Pakistan. PloS One 20, e0329735. doi: 10.1371/journal.pone.0329735 40839709 PMC12370205

[B40] Khan A. Swennen R. Panda S. K. Schoofs L. Luyten W. (2025b). Qualitative phytochemical analysis, thin-layer chromatographic profiling, and antimicrobial potential of banana cultivars. Electron. J. Biotechnol. 78, 64–75. doi: 10.1016/j.ejbt.2025.07.00542574925

[B41] Khatun S. Sen S. (2024). A comprehensive review on ethnomedicinal aspects, phytochemical and pharmacological properties of Limonia acidissima Linn. Pharmacogn. Res. 16, 688–697. doi: 10.5530/pres.16.4.8042362907

[B42] Kumar M. Dagar A. Gupta V. K. Sharma A. (2014). In silico docking studies of bioactive natural plant products as putative DHFR antagonists. Med. Chem. Res. 23, 810–817. doi: 10.1007/s00044-013-0654-930311153

[B43] Lalit M. Gangwal R. P. Dhoke G. V. Damre M. V. Khandelwal K. Sangamwar A. T. (2013). A combined pharmacophore modeling, 3D-QSAR and molecular docking study of substituted bicyclo-[3.3. 0] oct-2-enes as liver receptor homolog-1 (LRH-1) agonists. J. Mol. Struct. 1049, 315–325. doi: 10.1016/j.molstruc.2013.06.03542574925

[B44] Le N. T. M. Huy D. T. Le Minh T. T. Oanh V. T. T. (2019). Isolation and characterization of antibacterial compounds from Euphorbia tirucalli. Tạp chí Khoa học Công nghệ và Thực phẩm 18, 3–12.

[B45] Lee J. E. Jayakody J. T. M. Kim J. I. Jeong J. W. Choi K. M. Kim T. S. . (2024). The influence of solvent choice on the extraction of bioactive compounds from Asteraceae: A comparative review. Foods 13, 3151. doi: 10.3390/foods13193151 39410186 PMC11475975

[B46] Lee S. Y. So Y. J. Shin M. S. Cho J. Y. Lee J. (2014). Antibacterial effects of afzelin isolated from Cornus macrophylla on Pseudomonas aeruginosa, a leading cause of illness in immunocompromised individuals. Molecules 19, 3173–3180. doi: 10.3390/molecules19033173 24642906 PMC6271916

[B47] Lipinski C. A. (2004). Lead-and drug-like compounds: The rule-of-five revolution. Drug Discov. today: Technol. 1, 337–341. doi: 10.1016/j.ddtec.2004.11.007 24981612

[B48] Liu M. Veryser C. Lu J. G. Wenseleers T. De Borggraeve W. M. Jiang Z. H. . (2018). Bioassay-guided isolation of active substances from Semen Torreyae identifies two new anthelmintic compounds with novel mechanism of action. J. Ethnopharmacol. 224, 421–428. doi: 10.1016/j.jep.2018.06.026 29933012

[B49] Liu L. Yin Q. Dai Q. Chen X. Chen X. Wang J. B. . (2024). A new biphenanthrene with cytotoxic activity from Liparis nervosa (Thunb.) Lindl. Nat. Prod. Res. 38, 2012–2018. doi: 10.1080/14786419.2023.2233670 37661314

[B50] Londonkar R. L. (2016). *In vitro* cytotoxicity effect of kaempferol in breast cancer cell lines MCF-7 and lung cancer cell lines A459. Int. J. Curr. Microbiol. Appl. Sci. 5, 414–421. doi: 10.20546/ijcmas.2016.508.044

[B51] Lotha R. Sundaramoorthy N. S. Shamprasad B. R. Nagarajan S. Sivasubramanian A. (2018). Plant nutraceuticals (Quercetrin and Afzelin) capped silver nanoparticles exert potent antibiofilm effect against food borne pathogen Salmonella enterica serovar Typhi and curtail planktonic growth in zebrafish infection model. Microb. Pathogen. 120, 109–118. doi: 10.1016/j.micpath.2018.04.044 29715535

[B52] Maher C. Hassan K. A. (2023). The Gram-negative permeability barrier: tipping the balance of the in and the out. MBio 14, e01205-23. doi: 10.1128/mbio.01205-23 37861328 PMC10746187

[B53] Meunier M. Schinkovitz A. Derbré S. (2024). Current and emerging tools and strategies for the identification of bioactive natural products in complex mixtures. Nat. Prod. Rep. 41, 1766–1786. doi: 10.1039/d4np00006d 39291767

[B54] Nasim N. Sandeep I. S. Mohanty S. (2022). Plant-derived natural products for drug discovery: current approaches and prospects. Nucleus (Calcutta) 65, 399–411. doi: 10.1007/s13237-022-00405-3 36276225 PMC9579558

[B55] Nothias L. F. Nothias-Esposito M. Da Silva R. Wang M. Protsyuk I. Zhang Z. . (2018). Bioactivity-based molecular networking for the discovery of drug leads in natural product bioassay-guided fractionation. J. Nat. Prod. 81, 758–767. doi: 10.1021/acs.jnatprod.7b00737 29498278

[B56] Oliveira F. A. Pinto A. C. S. Duarte C. L. Taranto A. G. Lorenzato Junior E. Cordeiro C. F. . (2022). Evaluation of antiplasmodial activity in *silico* and *in vitro* of N-acylhydrazone derivatives. BMC Chem. 16, 50. doi: 10.1186/s13065-022-00843-9 35810303 PMC9271247

[B57] Padalia H. Chanda S. (2015). Antimicrobial efficacy of different solvent extracts of *Tagetes erecta* L. flower, alone and in combination with antibiotics. Appl. Microbiology: Open Access 1, 1–10. doi: 10.4172/2471-9315.1000106

[B58] Pagadala N. S. Syed K. Tuszynski J. (2017). Software for molecular docking: a review. Biophysical reviews, 9, 91–102. doi: 10.1007/s12551-016-0247-1 PMC5425816 28510083

[B59] Panda S. K. Padhi L. Leyssen P. Liu M. Neyts J. Luyten W. (2017). Antimicrobial, anthelmintic, and antiviral activity of plants traditionally used for treating infectious disease in the Similipal Biosphere Reserve, Odisha, India. Front. Pharmacol. 8, 658. doi: 10.3389/fphar.2017.00658 29109684 PMC5660100

[B60] Pandey S. Satpathy G. Gupta R. K. (2014). Evaluation of nutritional, phytochemical, antioxidant and antibacterial activity of exotic fruit “*Limonia acidissima*. J. Pharmacog. Phytochem. 3, 81–88.

[B61] Parvez G. M. M. Sarker R. K. (2021). Pharmacological potential of wood apple (*Limonia acidissima*): A review. Int. J. Minor Fruits Medicinal Aromatic Plants 7, 40–47. doi: 10.53552/ijmfmap.2021.v07ii02.003

[B62] Periferakis A. Periferakis K. Badarau I. Petran E. M. Popa D. C. Caruntu A. . (2022). Kaempferol: antimicrobial properties, sources, clinical, and traditional applications. Int. J. Mol. Sci. 23, 15054. doi: 10.3390/ijms232315054 36499380 PMC9740324

[B63] Pitchai P. Ulagi R. Mohan P. S. Gengan R. M. (2012). A novel alkaloid, four alkaloid precursors and a coumarin from *Feronia limonia*. Indian J. Chem. Section. B. (Online). 51, 1771–1775. doi: 10.1002/chin.20131519242587375

[B64] Qian C. D. Jiang F. S. Yu H. S. Shen Y. Fu Y. H. Cheng D. Q. . (2015). Antibacterial biphenanthrenes from the fibrous roots of *Bletilla striata*. J. Nat. Prod. 78, 939–943. doi: 10.1021/np501012n 25760525

[B65] Radziejewska I. Supruniuk K. Czarnomysy R. Buzun K. Bielawska A. (2021). Anti-cancer potential of afzelin towards AGS gastric cancer cells. Pharmaceuticals 14, 973. doi: 10.3390/ph14100973 34681197 PMC8539446

[B66] Rahman M. M. Gray A. I. (2002). Antimicrobial constituents from the stem bark of *Feronia limonia*. Phytochemistry 59, 73–77. doi: 10.1016/s0031-9422(01)00423-x 11754947

[B67] Romero N. Areche C. Cubides-Cárdenas J. Escobar N. García-Beltrán O. Simirgiotis M. . (2020). *In vitro* anthelmintic evaluation of *Gliricidia sepium*, *Leucaena leucocephala*, and *Pithecellobium dulce*: Fingerprint analysis of extracts by UHPLC-orbitrap mass spectrometry. Molecules 25, 3002. doi: 10.3390/molecules25133002 PMC7412154 32630065

[B68] Saleem B. A. Qurtam A. A. Shalabi A. R. Al-Zharani M. Abass K. S. Bräse S. . (2026). Design, synthesis, and mechanistic evaluation of novel pyrazole/thiazole chalcone hybrids as dual tubulin polymerization and COX-2 inhibitors with potent antiproliferative activity. RSC Adv. 16, 30052–30069. doi: 10.1039/d6ra03557d 42245077 PMC13231365

[B69] Sathasivampillai S. V. Sebastian P. R. (2021). Medicinal values of a food plant - *Limonia acidissima* Groff. BSJ Health Sci. 4, 240–245. doi: 10.19127/bshealthscience.861779

[B70] Schrödinger (2023). QikProp (New York, NY: Schrödinger, LLC).

[B71] Shao J. Zhang M. Wang T. Li Y. Wang C. (2016). The roles of CDR1, CDR2, and MDR1 in kaempferol-induced suppression with fluconazole-resistant Candida albicans. Pharm. Biol. 54, 984–992. 10.3109/13880209.2015.1091483 PMC11132302 26459663

[B72] Sharma P. Tenguria R. K. (2021). Phytochemical properties and health benefits of *Limonia acidissima*: A review. Int. Res. J. Plant Sci. 12, 1–6.

[B73] Sui J. Tang C. Ke C. Q. Ye Y. (2021). Dimeric 9, 10-dihydrophenanthrene derivatives from *Bletilla striata* and their atropisomeric nature. Fitoterapia 152, 104919. doi: 10.1016/j.fitote.2021.104919 33984433

[B74] Sujitha S. Venkatalakshmi P. (2021). Insights into the *In vitro* antioxidant, anti-inflammatory, and anticancer activities of *Limonia acidissima* fruits. Int. J. Life. Sci. Pharma Res. 11, 1–11. doi: 10.22376/ijpbs/lpr.2021.11.3.l1-1141064637

[B75] Tabaza Y. Z. Seong Z. K. Kim Y. M. Alshaer W. Aburjai T. A. (2024). Cytotoxicity of luteolin, a flavonoid compound isolated from *Anthemis palestina*. Trop. J. Pharm. Res. 23, 77–83. doi: 10.4314/tjpr.v23i1.10

[B76] Tatsimo S. J. N. Tamokou J. D. D. Havyarimana L. Csupor D. Forgo P. Hohmann J. . (2012). Antimicrobial and antioxidant activity of kaempferol rhamnoside derivatives from *Bryophyllum pinnatum*. BMC Res. Notes 5, 158. doi: 10.1186/1756-0500-5-158 22433844 PMC3353177

[B77] Tileklioğlu E. Aydın E. (2025). Investigation of the antiparasitic potential of luteolin: *In vitro* activity and comparison with standard therapeutics. Türkiye Parazitoloji Dergisi 49, 146–151. 10.4274/tpd.galenos.2025.52533<<< 41324231

[B78] Tran M. Truong P. C. H. Le T. T. T. Pham H. Bich V. N. T. Thien Y. N. H. . (2025). Revealing inhibitory activity of luteolin from Vietnamese *Jatropha podagrica* Hook against *Staphylococcus aureus* by integrating *in vitro* and in *silico* approaches. Sci. Rep. 15, 28537. doi: 10.1038/s41598-025-13655-3 40764363 PMC12325669

[B79] Warren G. L. Andrews C. W. Capelli A. M. Clarke B. LaLonde J. Lambert M. H. . (2006). A critical assessment of docking programs and scoring functions. J. Med. Chem. 49, 5912–5931. doi: 10.1021/jm050362n 17004707

[B80] Wei X. Wang W. Hu R. Gao X. Li B. Bai Y. . (2025). Advances in kaempferol: Extraction, biosynthesis, and application with antibacterial agents. Antibiotics 14, 1254. doi: 10.3390/antibiotics14121254 41463756 PMC12729929

[B81] Xie Y. Yang W. Tang F. Chen X. Ren L. (2015). Antibacterial activities of flavonoids: structure-activity relationship and mechanism. Curr. Med. Chem. 22, 132–149. doi: 10.2174/0929867321666140916113443 25245513

[B82] Xu J. Yu H. Qing C. Zhang Y. Liu Y. Chen Y. (2009). Two new biphenanthrenes with cytotoxic activity from *Bulbophyllum odoratissimum*. Fitoterapia 80, 381–384. doi: 10.1016/j.fitote.2009.05.007 19446615

[B83] Xue Z. Li S. Wang S. Wang Y. Yang Y. Shi J. . (2006). Mono-, bi-, and triphenanthrenes from the tubers of *Cremastra appendiculata*. J. Nat. Prod. 69, 907–913. doi: 10.1021/np060087n 16792409

[B84] Yaghoobi M. Moridi Farimani M. Khan A. Asadollahi M. Omrani M. Luyten W. . (2025). Investigation of phytochemical profiling and biological activities of methanol extract from *Eryngium billardieri*: antimicrobial, antibiofilm, and anthelmintic properties. Front. Plant Sci. 16, 1667335. doi: 10.3389/fpls.2025.1667335 41281318 PMC12631237

[B85] Yuan G. Guan Y. Yi H. Lai S. Sun Y. Cao S. (2021). Antibacterial activity and mechanism of plant flavonoids to gram-positive bacteria predicted from their lipophilicities. Sci. Rep. 11, 10471. doi: 10.1038/s41598-021-90035-7 34006930 PMC8131645

[B86] Zhou X. Zheng C. Gan L. Chen G. Zhang X. Song X. . (2016). Bioactive phenanthrene and bibenzyl derivatives from the stems of *Dendrobium nobile*. J. Nat. Prod. 79, 1791–1797. doi: 10.1021/acs.jnatprod.6b00252 27310249

