# Supplementary material for: Bioassay-guided isolation and structural characterization of antimicrobial phytoconstituents from Limonia acidissima L. leaves and evaluation of antimicrobial, anthelmintic, and cytotoxic effects: an in vitro and in silico approach
Source: Front Cell Infect Microbiol. 2026 Sep 11;16:1920478. doi: 10.3389/fcimb.2026.1920478 (PMC13612305; doi:10.3389/fcimb.2026.1920478)
Supplement: Supplementary file 1 [file DataSheet1.docx]

**SUPPLEMENTARY MATERIAL**

**Bioassay-guided isolation and structural characterization of antimicrobial phytoconstituents from *Limonia acidissima* L. leaves and evaluation of antimicrobial, anthelmintic, and cytotoxic effects: an *in vitro* and *in silico* approach**

Sujogya Kumar Panda^1,2^*†, Ajmal Khan^1,3^*†, Masoud Besati^4^, Mahdi Yaghoobi^1^, Haibo Hu^1,5^, Jan Paeshuyse^6^, Liliane Schoofs^1^, Walter Luyten^1^

^1^Department of Biology, Animal Physiology and Neurobiology Section, KU Leuven, Leuven, Belgium

^2^Centre for Biotechnology, School of Pharmaceutical Sciences, Siksha ‘O’ Anusandhan (Deemed to be University), Kalinga Nagar, Bhubaneswar-751003, India

^3^Department of Zoology, University of Swat, Charbagh Swat, Khyber Pakhtunkhwa, Pakistan

^4^Department of Pharmaceutical Engineering, Medicinal Plants and Drugs Research Institute, Shahid Beheshti University, Evin, Tehran, Iran

^5^National Engineering Research Center for Modernization of Traditional Chinese Medicine - Hakka Medical Resources Branch, School of Pharmacy, Gannan Medical University, 341000 Ganzhou, China

^6^Department of Biosystems, Animal and Human (A2H), KU Leuven, Leuven, Belgium

*Corresponding author(s). E-mails: [sujogyapanda@soa.ac.in](mailto:sujogyapanda@soa.ac.in); [ajmalkhan399@hotmail.com](mailto:ajmalkhan399@hotmail.com);

*Contributing author(s): [masoud.besati@gmail.com](mailto:masoud.besati@gmail.com); [m_yaghoobi@yahoo.com](mailto:m_yaghoobi@yahoo.com); [hhb2017@gmu.edu.cn](mailto:hhb2017@gmu.edu.cn)**;** [jan.paeshuyse@kuleuven.be](mailto:jan.paeshuyse@kuleuven.be); [liliane.schoofs@kuleuven.be](mailto:liliane.schoofs@kuleuven.be); [walter.luyten@kuleuven.be](mailto:walter.luyten@kuleuven.be)

**^†^**These authors contributed equally to this work and share first authorship.

SUPPLEMENATRY TABLES:

**Table S1**: HPLC gradient conditions for the selected fractions of *L. acidissima* leaves.

| Fractions | Gradients |
| --- | --- |
| F42 | 0-5 min. 30% B, 5-35 min. 60% B, 35-45 min. 80% B, 45-55 min. 100% B, 55-60 min. 100% B. |
| F105 | 0-5 min. 20% B, 5-35 min. 25% B, 35-45 min. 50% B, 45-55 min. 100% B, 55-60 min. 100% B. |
| F124 | 0-5 min. 10% B, 5-35 min. 30% B, 35-45 min. 70% B, 45-55 min. 100% B, 55-60 min. 100% B. |
| F169 | 0-5 min. 5% B, 5-35 min. 30% B, 35-45 min. 70% B, 45-55 min. 100% B, 55-60 min. 100% B. |

**Table S2:** Interactions and scores of components and receptors.

| No. | Name of  Compound | Scores | | Interactions | | | Amino acids | 2D Interactions |
| --- | --- | --- | --- | --- | --- | --- | --- | --- |
|  |  | XPG Score/ Docking Score | Glide Emodel | H-bonds | π-π bonds | Salt bridges |  |  |
| 1CX2 (A549) | | |  |  |  |  |  |  |
| 1 | Kaempferol-3-*O*-alpha-L-rhamnoside | -7.751 | -34.744 | 1 (One)  Tyr385-OH | - | - | Leu531, Ser530, Ala527, Gly526, Val526, Met522, Phe518, Ile517, Ala516, Arg513, Gln192, Tyr355, Ser353, Leu352, Val349, Tyr348 |  |
| 2 | Luteolin | -7.061 | -55.526 | 1 (One)  Arg120-OH | - | - | Leu531, Ser530, Ala527, Val116, Leu359, Arg120, Arg513, Ala516, Ile517, Phe518, Tyr355, Ser353, Leu352, Val349 |  |
| 3 | Kaempferol | -6.724 | -53.108 | - | - | - | Met113, Val116, Arg120, Leu531, Ser530, Ala527, Val349, Leu352, Ser353, Tyr355, Phe518, Phe518, Ile517, Ala516 |  |
| 4 | 2,2′-dihydroxy-4,7,4′,7′-tetramethoxy-1,1′-biphenanthrene | 0.055 | 0 | - | - | - | Lys492 |  |
| 1TV5 (*Pf-*DHFR) | | |  |  |  |  |  |  |
| 1 | Kaempferol-3-*O*-alpha-L-rhamnoside | -9.375 | 3.081 | 2(Two)  Lys429-O, HO-Asn458 | 1(One)  Tyr528 | - | Ser529, Tyr528, Ile263, Ile272, Asn274, Cys276, Phe278, Lys429, Ser505, Gly506, Gly507, Ile508 |  |
| 2 | Kaempferol | -8.625 | -60.19 | 2 (Two)  HO-Ser457, HOAsn458 | 2 (Two)  Tyr528 | - | Leu481, Gly478, Ser477, Ser529, Tyr528, Ile272, Asn274, Cys276, Lys229, Gly226, Ala225, Thr459, Asn458, Ser457 |  |
| 3 | Luteolin | -8.413 | -54.654 | 1 (One)  HO-Ser457 | 2(Two)  Tyr528 | - | Leu481, Gly478, Ser477, Ser529, Tyr528, Ile272, Asn274, Cys276, Lys229, Gly226, Ala225, Thr459, Asn458, Ser457 |  |
| 4 | 2,2′-dihydroxy-4,7,4′,7′-tetramethoxy-1,1′-biphenanthrene | - | - | - | - | - | - | - |
| 2Y2H (*S. aureus*) | | |  |  |  |  |  |  |
| 1 | Kaempferol | -5.994 | -52.442 | 1 (One)  HO-Met556 | 2 (Two)  Tyr498 | - | Ala499, Tyr498, Met497, Gly656, Thr654, Gly653, Ser516, Asn518, Met556, Ala459, Ser460, Lyn463 |  |
| 2 | Luteolin | -5.701 | -48.562 | 1 (One)  HO-Met556 | 2 (Two)  Tyr498 | - | Ala499, Tyr498, Met497, Gly656, Thr654, Gly653, Ser516, Asn518, Met556, Ala459, Ser460, Lyn463 |  |
| 3 | Kaempferol-3-*O*-alpha-L-rhamnoside | -5.457 | -39.103 | 4 (Four)  Asn518-O, Asn518-OH, HO-Met556, Gly656-OH | 2 (Two)  Tyr498 | - | Asn518, Met556, Gln657, Gly656, Thr655, Thr654, Met497, Tyr498, Ala499 |  |
| 4 | 2,2′-dihydroxy-4,7,4′,7′-tetramethoxy-1,1′-biphenanthrene | 0.350 | 0.027 | - | - | - | - |  |
| 3PHC (WI-26 VA4) | | |  |  |  |  |  |  |
| 1 | Luteolin | -7.913 | -45.193 | 3 (Three)  HO-Met159, HO-His7, HO-PO4502 | - | - | Trp212, Pro209, Asp206, Val73, His7, Arg45, Leu170, Phe217, Ser157, Met159, Met159, Tyr160, Val181, Glu182, Met183, Glu184, Gly23 |  |
| 2 | Kaempferol-3-*O*-alpha-L-rhamnoside | -7.784 | 61.344 | 5 (Five)  HO-Met159, Tyr160-O, PO4502-OH, HO-Asp206. His7-OH | 1 (One)  Tyr160 | - | Phe217, Asn219, Ser157, Met159, Met159, Tyr160, Val181, Glu182, Met183, Glu184, Arg88, Ser91, Cys92, Gly93, Asp206, Pro209, Trp212 |  |
| 3 | Kaempferol | -7.752 | -43.938 | 2 (Two)  HO-Met159, HO-PO4502 | 2 (Two)  Tyr160 | - | Ser157, Met159, Tyr160, Val181, Glu182, Met183, Glu184, Trp212, Pro209, Asp210, Gly93, |  |
| 4 | 2,2′-dihydroxy-4,7,4′,7′-tetramethoxy-1,1′-biphenanthrene | -0.085 | 0 | 1 (One)  Gly124-O | - | - | Hie123126, Arg116, Arg169 |  |
| 5TZ1 (*C. albicans*) | | |  |  |  |  |  |  |
| 1 | Luteolin | -9.655 | -58.755 | 1 (One)  HO-Met508 | Tyr118 | - | Gly307, Gly308, Thr311, Met508, Val509, Ile131, Tyr132, Thr122, Leu121, Phe126, Tyr118, Phe380 |  |
| 2 | Kaempferol-3-*O*-alpha-L-rhamnoside | -9.387 | 18.484 | 2 (Two)  HO-Tyr132 | 1 (One)  Phe228 | - | Gly307, Gly308, Thr311, Met508, Val509, Ile131, Tyr132, Thr122, Leu121, Phe126, Tyr118, Phe380, Hip310, Ile379, Ser378, Hie377, Leu376 |  |
| 3 | Kaempferol | -9.308 | -53.465 | 2 (Two)  HO-Tyr132, HO-Met508 | - | - | Ile131, Tyr132, Thr122, Leu121, Tyr118, Pro230, Phe228, Met508, Val509, Leu376, Thr311 |  |
| 4 | 2,2′-dihydroxy-4,7,4′,7′-tetramethoxy-1,1′-biphenanthrene | -7.743 | 47.817 | 2 (Two)  His377-O, HO-Ser378 | 2 (Two)  Tyr118, His377 | - | Leu376, His377, Ser378, Ile379, Phe380, Val509, Met508, Ser507, Ser506, Tyr505, Tyr64, Ala61 |  |
| 6F86 (*E. coli)* | | |  |  |  |  |  |  |
| 1 | Luteolin | -6.367 | -54.394 | 1 (One)  HO-Asp73 | - | - | Pro79, Ile78, Gly77, Arg76, Gly75, Asp73, Glu50, Ala47, Asn46 |  |
| 2 | Kaempferol | -5.567 | -48.039 | - | - | - | Pro79, Ile78, Gly77, Arg76, Gly75, Asp73, Glu50, Ala47, Asn46 |  |
| 3 | Kaempferol-3-*O*-alpha-L-rhamnoside | -5.097 | -62.692 | 2 (Two)  HO-Asp49, HO-Asp73 | - | - | Pro79, Ile78, Gly77, Arg76, Gly75, Asp73, Glu50, Ala47, Asn46 |  |
| 4 | 2,2′-dihydroxy-4,7,4′,7′-tetramethoxy-1,1′-biphenanthrene | -1.813 | -39.904 | - | - | - | Pro79, Ile78, Gly77, Arg76, Gly75, Asp73, Glu50, Ala47, Asn46, Val43, Asp49, Glu50, Ile94 |  |
| 6E88 (*C. elegans*) | | |  |  |  |  |  |  |
| 1 | Kaempferol-3-*O*-alpha-L-rhamnoside | -9.274 | -80.032 | 4 (Four)  HO-Asn226, HC-Ser138, HO-Asn99 | - | - | Asn204, Ile169, Pro171, Ser138, Phe139, Gly140, Gly141, Gly142, Thr143, Gly144, Asn226, Gly10, Gln11, Ala12, Gly13 |  |
| 2 | Luteolin | -8.866 | -66.247 | 4 (Four)  Phe139-OH, HO-Ile169, HO-Asn226 | - | - | Tyr222, Gln11, Ala12, Asn226, Gln15, Ile16, Ile169, Pro171, Ser138, Phe139, Gly140, Asn204 |  |
| 3 | Kaempferol | -8.051 | -53.247 | 4 (Four)  HO-Glu181, HO-Ile169, Phe139-OH, HO-Asn226 | - | - | Tyr222, Asn204, Ser176, Thr177, Ala178, Glu181, Pro171, Tyr170, Ile169, Gly140, Phe139, Ser138 |  |
| 4 | 2,2′-dihydroxy-4,7,4′,7′-tetramethoxy-1,1′-biphenanthrene | -0.767 | -69.333 | 2 (Two)  Thr177-O^-^, HO-Glu181 | 1 (One)  Tyr222 | - | Ile229, Asn226, Leu225, Tyr222, Ser176, Thr177, Ala178, Glu181, Ser138, Gly140, Gly141, Thr143, Asp96, Ala97, Asn99 |  |

**Table S3**:Antimicrobial, anthelmintic, and cytotoxicity assessment of 4 compounds isolated from *L. acidissima*.

| **Compounds** | **Antibacterial activity** | | | | **Antifungal activity** | | **Anthelmintic activity** | | **Cytotoxicity** | | | |
| --- | --- | --- | --- | --- | --- | --- | --- | --- | --- | --- | --- | --- |
|  | ***S. aureus*** | | ***E. coli*** | | ***C. albicans*** | | ***C. elegans*** | | **A549** | | **WI-26 VA4** | |
|  | **IV±SD**  (50 µg/mL) | **IC_50_** | **IV±SD**  (50 µg/mL) | **IC_50_**  (95% CI) | **IV±SD**  (20 µg/mL) | **IC_50_**  (95% CI) | **IV±SD**  (5 µg/mL) | **IC_50_**  (95% CI) | **IV±SD**  (10 µg/mL) | **IC_50_**  (95% CI) | **IV±SD**  (10 µg/mL) | **IC_50_**  (95% CI) |
| **2,2′-dihydroxy-4,7,4′,7′-tetramethoxy-1,1′-biphenanthrene** | 76±2.1 | 19 (12.9−30.5) | 39±3.5 | 64 (29.2−86.7) | 30±1.4 | 33 (17.9−55.4) | 34±2.1 | 7 (4.2−21.5) | 19±1.4 | 35 (14.2−66.5) | 32±3.5 | 16  (9.3−97.4) |
| **Kaempferol** | 85±3.5 | 17 (11.6−25.9) | 50±2.8 | 28 (16.1−77.4) | 61±2.1 | 10 (6.7−20.7) | 26±2.8 | 9 (4.7−18.2) | 24±1.4 | 33 (14.2−58.1) | 15±0.7 | 42 (24.0−95.9) |
| **Luteolin** | 73±2.8 | 19 (12.9−32.2) | 48±2.8 | 44 (21.9−76.6) | 57±1.4 | 16 (14.0−20.3) | 38±5.6 | 8 (4.9−48.4) | 44±3.5 | 9 (5.8−30.1) | 26±3.5 | 24 (14.8−75.7) |
| **Kaempferol-3-*O*-alpha-L-rhamnoside** | 61±4.2 | 22 (16.7−33.1) | 53±2.1 | 35 (23.5−72.8) | 37±2.8 | 32 (22.8−69.1) | 29±3.5 | 10 (5.9−32.0) | 20±1.4 | 20 (11.8−27.5) | 14±2.1 | 33 (17.3−82.6) |
| *P | 95±2 | 0.28 | 94±2 | 0.02 | 94±3 | 0.01 | 98±2 | 1.6 | 82±2 | ---- | 89±3 | ---- |

Legends: IV- inhibition value; SD- standard deviation, IC_50_- 50% inhibitory concentration, CI- confidence interval

*P: Positive Control: Ciprofloxacin for *S. aureus* and *E.coli*; miconazole for *C. albicans*; levamisole for *C. elegans*; gossypol for A549 and WI 26 VA4 cell lines.

**SUPPLEMENTARY FIGURES:**

**Figure S1:** Chromatogram of silica gel fractions of the acetone extracts of *L. acidissima* leaves recorded at 254 nm. The x-axis represents retention time in minutes.

% inhibition

| Frcations | 1-fold | 2-fold | 4-fold | 8-fold | 16-fold | 32-fold |
| --- | --- | --- | --- | --- | --- | --- |
| F41 | 100 | 100 | 91 | 65 | 30 | 5 |
| F42 | 100 | 100 | 98 | 91 | 44 | 9 |
| F43 | 100 | 90 | 88 | 74 | 41 | -2 |
| F56 | 98 | 91 | 75 | 55 | 34 | -3 |
| F57 | 100 | 97 | 88 | 66 | 51 | 13 |
| F58 | 100 | 88 | 71 | 55 | 40 | 3 |
| F104 | 96 | 80 | 69 | 21 | -1 | -6 |
| F105 | 98 | 87 | 76 | 28 | 26 | 7 |
| F106 | 95 | 84 | 70 | 18 | 6 | -5 |
| F123 | 94 | 70 | 54 | 40 | 8 | -4 |
| F124 | 100 | 76 | 64 | 48 | 14 | -3 |
| F125 | 96 | 71 | 61 | 34 | 6 | -8 |
| F131 | 94 | 77 | 62 | 22 | 3 | -9 |
| F132 | 100 | 83 | 70 | 26 | 18 | 4 |
| F133 | 100 | 80 | 56 | 25 | 9 | -6 |
| F168 | 90 | 57 | 42 | 25 | 7 | -5 |
| F169 | 92 | 63 | 41 | 33 | 1 | -2 |
| F170 | 89 | 60 | 37 | 22 | -3 | -7 |

**Figure S2:** Antimicrobial activity of serially diluted consecutive active fraction clusters of *L. acidissima* against *S. aureus*.

A)

LA-42

|  |  |
| --- | --- |
| 1 | 5 |
| 2 | 2 |
| 3 | 4 |
| 4 | 2 |
| 5 | 8 |
| 6 | 2 |
| 7 | 7 |
| 8 | -4.8041 |
| 9 | 12 |
| 10 | 10 |
| 11 | 14 |
| 12 | 3 |
| 13 | 1 |
| 14 | 1 |
| 15 | 1.6752 |
| 16 | 6 |
| 17 | 11 |
| 18 | 12 |
| 19 | 5 |
| 20 | 13 |
| 21 | 6.05012 |
| 22 | 13 |
| 23 | 19 |
| 24 | 16 |
| 25 | 22 |
| 26 | 9 |
| 27 | 14 |
| 28 | 1.49522 |
| 29 | 16 |
| 30 | 20 |
| 31 | 7 |
| 32 | 3.80728 |
| 33 | 13 |
| 34 | 8 |
| 35 | 12 |
| 36 | 4 |
| 37 | 3.90419 |
| 38 | 5.69016 |
| 39 | 3.41963 |
| 40 | 1 |
| 41 | 14 |
| 42 | 10 |
| 43 | 17 |
| 44 | 4.25031 |
| 45 | 6.43777 |
| 46 | 4.45798 |
| 47 | 5.71785 |
| 48 | 19 |
| 49 | 11 |
| 50 | 2.92122 |
| 51 | 3.19812 |
| 52 | 15 |
| 53 | 19 |
| 54 | 86 |
| 55 | 32 |
| 56 | 12 |
| 57 | 8 |
| 58 | 3 |
| 59 | 5 |
| 60 | 2 |

B)

**Figure S3:** Semi-preparative HPLC chromatogram of Fraction 42 of L. acidissima (A) and antimicrobial activity (% inhibition) of the resulting HPLC subfractions against S. aureus (B).

Note: a) HPLC chromatogram (black = UV absorbance at 190 nm; plum = UV absorbance at 254 nm; light blue = acetonitrile (ACN) concentration gradient (%)).

A)

LA-105

|  |  |
| --- | --- |
| 1 | 2 |
| 2 | 0 |
| 3 | 4.40463 |
| 4 | 2.80172 |
| 5 | 8 |
| 6 | 4 |
| 7 | 11 |
| 8 | 4 |
| 9 | 20.5011 |
| 10 | 17 |
| 11 | 21.9962 |
| 12 | 15.3556 |
| 13 | 8 |
| 14 | 16.7295 |
| 15 | 13.4294 |
| 16 | 2.03394 |
| 17 | 20.8648 |
| 18 | 31 |
| 19 | 20.2856 |
| 20 | 28 |
| 21 | 23 |
| 22 | 7 |
| 23 | 9 |
| 24 | 12 |
| 25 | 29 |
| 26 | 12.7963 |
| 27 | 9.60399 |
| 28 | 6.29041 |
| 29 | 17 |
| 30 | 7.96067 |
| 31 | 13 |
| 32 | 24 |
| 33 | 18 |
| 34 | 26 |
| 35 | 22 |
| 36 | 7.11207 |
| 37 | 9 |
| 38 | 16 |
| 39 | 9.52317 |
| 40 | 6 |
| 41 | 3 |
| 42 | -1 |
| 43 | 3 |
| 44 | 88 |
| 45 | 17 |
| 46 | 10.695 |
| 47 | 19 |
| 48 | 4 |
| 49 | -2.55927 |
| 50 | 5.76509 |
| 51 | 5.54957 |
| 52 | 4.78179 |
| 53 | 14 |
| 54 | 21 |
| 55 | 7 |
| 56 | -12.6347 |
| 57 | 3 |
| 58 | -0.673491 |
| 59 | 15.0862 |
| 60 | -9.99461 |

B)

**Figure S4:** Semi-preparative HPLC chromatogram of Fraction 105 of L. acidissima (A) and antimicrobial activity (% inhibition) of the resulting HPLC subfractions against S. aureus (B).

Note: a) HPLC chromatogram (black = UV absorbance at 190 nm; plum = UV absorbance at 254 nm; light blue = acetonitrile (ACN) concentration gradient (%)).

A)

LA-124

|  |  |
| --- | --- |
| 1 | 4.94331 |
| 2 | 2 |
| 3 | 8.34467 |
| 4 | -2 |
| 5 | 19.1497 |
| 6 | 12 |
| 7 | 17.3469 |
| 8 | -2 |
| 9 | 2.55102 |
| 10 | 14 |
| 11 | 10.6576 |
| 12 | 3.84354 |
| 13 | 10.6122 |
| 14 | 5 |
| 15 | 13.1179 |
| 16 | 14.059 |
| 17 | 7.5737 |
| 18 | 9 |
| 19 | -3 |
| 20 | 1 |
| 21 | 12.2676 |
| 22 | 6 |
| 23 | -3 |
| 24 | 31 |
| 25 | 22 |
| 26 | 8.70748 |
| 27 | 3 |
| 28 | 8.93424 |
| 29 | 12.3583 |
| 30 | 12.449 |
| 31 | 91 |
| 32 | 5 |
| 33 | 1.00907 |
| 34 | 4.9093 |
| 35 | 7 |
| 36 | 10.0454 |
| 37 | 2 |
| 38 | 7 |
| 39 | 10.1247 |
| 40 | -2.74376 |
| 41 | 4.31973 |
| 42 | 5.12472 |
| 43 | 10.9297 |
| 44 | 12.8118 |
| 45 | 12.381 |
| 46 | 10.2154 |
| 47 | 11.5306 |
| 48 | -6.39456 |
| 49 | -3.18594 |
| 50 | 8.37868 |
| 51 | 10.5782 |
| 52 | 8.79819 |
| 53 | 8.34467 |
| 54 | 15 |
| 55 | 11 |
| 56 | -4 |
| 57 | -5.90703 |
| 58 | 0.544218 |
| 59 | 3.85488 |
| 60 | 4.95465 |

B)

**Figure S5:** Semi-preparative HPLC chromatogram of Fraction 124 of L. acidissima (A) and antimicrobial activity (% inhibition) of the resulting HPLC subfractions against S. aureus (B).

Note: a) HPLC chromatogram (black = UV absorbance at 190 nm; plum = UV absorbance at 254 nm; light blue = acetonitrile (ACN) concentration gradient (%)).

A)

LA-169

|  |  |
| --- | --- |
| 1 | 2 |
| 2 | 0 |
| 3 | 4 |
| 4 | -1 |
| 5 | 17.1355 |
| 6 | 2 |
| 7 | -4 |
| 8 | -3 |
| 9 | 6 |
| 10 | 21 |
| 11 | 12 |
| 12 | -5 |
| 13 | -11 |
| 14 | 16 |
| 15 | 27 |
| 16 | 26.2169 |
| 17 | 1 |
| 18 | 18.9798 |
| 19 | 6 |
| 20 | 26.5904 |
| 21 | 2 |
| 22 | 3 |
| 23 | -2 |
| 24 | -6 |
| 25 | 3 |
| 26 | 7 |
| 27 | 6 |
| 28 | 5 |
| 29 | 15.8749 |
| 30 | 19 |
| 31 | -3 |
| 32 | 10 |
| 33 | 9.78172 |
| 34 | 75 |
| 35 | 13.6337 |
| 36 | 2 |
| 37 | 7 |
| 38 | 13.6571 |
| 39 | 2 |
| 40 | 16 |
| 41 | 6.02311 |
| 42 | 8.70783 |
| 43 | 10 |
| 44 | 3 |
| 45 | 15.0928 |
| 46 | 4 |
| 47 | 8.99965 |
| 48 | -0.151745 |
| 49 | 5.80133 |
| 50 | 2 |
| 51 | -6 |
| 52 | 2 |
| 53 | 17 |
| 54 | 4.12046 |
| 55 | -11.3459 |
| 56 | -2.20614 |
| 57 | 0.723707 |
| 58 | 8.40434 |
| 59 | 1 |
| 60 | 6 |

B)

**Figure S6.** Semi-preparative HPLC chromatogram of Fraction 169 of L. acidissima (A) and antimicrobial activity (% inhibition) of the resulting HPLC subfractions against S. aureus (B).

Note: a) HPLC chromatogram (black = UV absorbance at 190 nm; plum = UV absorbance at 254 nm; light blue = acetonitrile (ACN) concentration gradient (%)).

MS^1^

MS^2^

**Figure S7:** HRMS spectra (MS^1^ and MS^2^) of Compound 1: 2,2′-dihydroxy-4,7,4′,7′-tetramethoxy-1,1′-biphenanthrene.

MS^1^

MS^2^

**Figure S8:** HRMS spectra (MS^1^ and MS^2^) of Compound 2: kaempferol.

**MS^1^**

**MS^2^**

**Figure S9:** HRMS spectra (MS^1^ and MS^2^) of Compound 3: luteolin.

**MS^1^**

**MS^2^**

**Figure S10:** HRMS spectra (MS^1^ and MS^2^) of Compound 4: kaempferol‑3‑O‑α‑L‑rhamnopyranoside or afzelin.

**Figure S11.** NMR spectrum of Compound 4: kaempferol‑3‑*O*‑α‑L‑rhamnopyranoside.
